# Comparative transcriptome and methylome of polar bears, giant and red pandas reveal diet‐driven adaptive evolution

**DOI:** 10.1111/eva.13731

**Published:** 2024-06-17

**Authors:** Lei Chen, Jinnan Ma, Wencai Xu, Fujun Shen, Zhisong Yang, Christian Sonne, Rune Dietz, Linzhu Li, Xiaodie Jie, Lu Li, Guoqiang Yan, Xiuyue Zhang

**Affiliations:** ^1^ Key Laboratory of bio‐Resources and eco‐Environment, Ministry of Education, College of Life Science Sichuan University Chengdu China; ^2^ College of Continuing Education Yunnan Normal University Kunming China; ^3^ Sichuan Key Laboratory for Conservation Biology of Endangered Wildlife Chengdu Research Base of Giant Panda Breeding Chengdu China; ^4^ Sichuan Academy of Giant Panda Chengdu China; ^5^ Arctic Research Centre, Faculty of Science and Technology, Department of Ecoscience Aarhus University Roskilde Denmark; ^6^ Sichuan Key Laboratory of Conservation Biology on Endangered Wildlife, College of Life Sciences Sichuan University Chengdu China

**Keywords:** adaptive evolution, giant panda, methylome, polar bear, red panda, transcriptome

## Abstract

Epigenetic regulation plays an important role in the evolution of species adaptations, yet little information is available on the epigenetic mechanisms underlying the adaptive evolution of bamboo‐eating in both giant pandas (*Ailuropoda melanoleuca*) and red pandas (*Ailurus fulgens*). To investigate the potential contribution of epigenetic to the adaptive evolution of bamboo‐eating in giant and red pandas, we performed hepatic comparative transcriptome and methylome analyses between bamboo‐eating pandas and carnivorous polar bears (*Ursus maritimus*). We found that genes involved in carbohydrate, lipid, amino acid, and protein metabolism showed significant differences in methylation and expression levels between the two panda species and polar bears. Clustering analysis of gene expression revealed that giant pandas did not form a sister group with the more closely related polar bears, suggesting that the expression pattern of genes in livers of giant pandas and red pandas have evolved convergently driven by their similar diets. Compared to polar bears, some key genes involved in carbohydrate metabolism and biological oxidation and cholesterol synthesis showed hypomethylation and higher expression in giant and red pandas, while genes involved in fat digestion and absorption, fatty acid metabolism, lysine degradation, resistance to lipid peroxidation and detoxification showed hypermethylation and low expression. Our study elucidates the special nutrient utilization mechanism of giant pandas and red pandas and provides some insights into the molecular mechanism of their adaptive evolution of bamboo feeding. This has important implications for the breeding and conservation of giant pandas and red pandas.

## 1 INTRODUCTION

Adaptive evolution is a dynamic process by which species or populations become better suited to their environment through natural selection mechanisms (Edelaar et al., 2023). Organisms respond to environmental pressures like food or climate change by constantly adjusting their phenotype, making their phenotypic traits more favourable for survival in complex environments. Over time, natural selection acts on these variations to shape the population, favouring individuals with the highest fitness and most adaptive phenotypes and genotypes (Cooper, 2017). Diet‐driven adaptive evolution represents a significant aspect of how environmental pressures can shape the genetic and phenotypic landscape of species. Various instances illustrate how dietary preferences and constraints have been pivotal in determining the evolutionary paths of species. For example, Darwin's finches exhibit a remarkable variety of beak shapes and sizes, each adapted to specific feeding habits, ranging from seed‐eating to insect‐hunting (Grant & Grant, 2006). Similarly, the evolution of lactase persistence in human populations is a direct response to dairy consumption, showcasing how dietary practices can influence genetic adaptations (Tishkoff et al., 2007). Giant pandas (*Ailuropoda melanoleuca*) and red pandas (*Ailurus fulgens*) are ideal models for studying diet‐driven adaptive evolution. Despite belonging to Carnivora, they have both transitioned from a carnivorous diet to a specialized bamboo diet, a remarkable shift that has led to the emergence of convergent adaptive features. Notably, both species have developed pseudothumbs, enhancing their ability to grasp bamboo efficiently, a morphological convergence that underscores the profound impact of dietary specialization on evolutionary trajectories (Hu et al., 2017). This adaptation is paralleled by molecular convergences, such as the pseudogenization of taste receptor genes, the evolution of digestive enzymes, and adaptations of the gut microbiome to bamboo cellulose digestion and cyanide detoxification (Hu et al., 2017; Huang et al., 2018, 2021; Li et al., 2010, 2015; McKenney et al., 2018; Zhang et al., 2018; Zhu et al., 2018). Extensively documented, the shared dietary specialization of giant and red pandas in bamboo, despite their belonging to different families, highlights convergent evolutionary outcomes driven by identical ecological pressures (Hu et al., 2017; Roberts & Gittleman, 1984; Wei et al., 1999). This offers a unique perspective on how environmental factors shape evolutionary paths across disparate lineages. However, while significant focus has been placed on the morphological, genomic, and gut microbiome adaptations of these species to a bamboo diet, the exploration of gene expression and epigenetic regulation in the context of their dietary shifts has yet to be fully comprehensive.

DNA methylation is a stable and heritable epigenetic modification that can regulate gene expression without changing the DNA nucleotide sequence (Goldberg et al., 2007; Heard & Martienssen, 2014; Kim & Costello, 2017; Law & Jacobsen, 2010; Takahashi et al., 2023). Alterations in DNA methylation patterns, as a response to environmental signals, can regulate gene expression and enable organisms to acclimate to environmental changes (Anderson et al., 2012; Liu, 2013; Thiebaut et al., 2019; Waterland, 2006). In addition, specific environmental or evolutionary pressures on mammals may, to some extent, lead to the conservation of methylation patterns across generations, contributing to adaptive responses (Daxinger & Whitelaw, 2012; Fitz‐James & Cavalli, 2022; Legoff et al., 2019). Comparative methylome studies within and between species have revealed that many adaptive phenotypic changes result from changes in DNA methylation and subsequent regulation (Boyko et al., 2010; Kucharski et al., 2008; Thorson et al., 2017). Thus, conducting genome‐wide methylation profiling of giant and red pandas may provide insight into the adaptive evolutionary mechanisms involved in their bamboo consumption.

Yizhen et al. (2023) compared the patterns of change in liver transcriptomes from no‐feeding to suckling and then to adulthood in animals with different diets, such as giant pandas, red pandas, mice, and ferrets, respectively. They found that, during dietary transitions, both red pandas and giant pandas exhibit similar changes in gene expression patterns related to nutrient metabolism. Additionally, the changes in carbohydrate digestion and absorption observed in red and giant pandas were similar to those seen in herbivores and omnivores (Yizhen et al., 2023). Conversely, the changes in fat digestion and absorption in red and giant pandas significantly diverge from those observed in the carnivorous ferret. The research identified specific adaptive gene expression traits linked to digestive metabolism in both giant pandas and red pandas, driven by their shared diet. However, including omnivores in this comparative analysis may obscure some characteristics pertinent to the bamboo diet. Furthermore, there has been a lack of comparative studies involving a pivotal species within the Carnivora order, notably the polar bear (*Ursus maritimus*), which has a closer phylogenetic relationship with the giant panda yet has evolved distinct dietary preferences, specializing as a carnivore (Liu et al., 2014). Given the critical role of the liver in digestion and metabolism, acting as a central hub in processing, converting, storing, and regulating nutrients and energy sources within the body, its functions are various and essential for maintaining overall health and metabolic balance (Adeva‐Andany et al., 2016; Grune et al., 1995; Nguyen et al., 2008; Robinson et al., 2016; Rosebrough et al., 1988; Rui, 2014). Therefore, polar bears were introduced in this study and a comprehensive cross‐species comparative analysis of transcriptome and methylome was performed on the livers of giant pandas, red pandas, and polar bears. This study can help to better understand the unique convergent nutrient utilization mechanisms of giant pandas and red pandas. This is also of great significance for the breeding and conservation of giant and red pandas.

## 2 MATERIALS AND METHODS

### 2.1 Animal sample collection

All liver tissue samples from giant pandas and red pandas were provided by the Chengdu Research Base of Giant Panda Breeding and the China Research and Conservation Centre for Giant Panda at Dujiangyan, Sichuan Province, China. Samples from polar bears were provided by Aarhus University. All polar bear samples were legally exported from Greenland into Denmark (IUCN CITES: 18GL1717224/19GL1717365) and imported into Denmark (IM 0928‐712/18/IM0816‐496/19). These activities received approval from the Ethics Committee of the College of Life Sciences, Sichuan University (approval number: 20190506001). All animals sampled had died from natural causes or accidents, unrelated to the design of this experiment or the sample collection. All livers sampled were normal in morphology and size, and none showed pathological changes. The excised liver tissue was immediately stored in a −80°C refrigerator.

### 2.2 RNA library preparation, sequencing, and data processing

Comparative transcriptome analysis included 10 samples from three species, of which two polar bear samples require RNA library preparation and sequencing, and the other four giant panda samples and four red panda samples have already undergone library preparation and sequencing in our previous study (Yizhen et al., 2023). Liver tissues involved in transcriptome sequencing were dissected and homogenized, and total RNA was extracted according to the instructions for use of TRIzol reagent (Invitrogen, Carlsbad, CA, USA). Samples with RNA integrity number (RIN) > 5.8 were used for the next step of RNA sequencing (RNA‐seq) library construction. Library construction was performed according to the NEBNext® UltraTM RNA Library PrepKit for Illumina® (NEB, USA) instructions. After column purification, the quality of the resulting libraries was assessed on an Agilent Bioanalyzer 2100 system. The prepared libraries were sequenced on the Illumina NovaSeq 6000 platform. All reads obtained have been submitted to NCBI Sequence Read Archive with BioProject number PRJNA865071. Table 1 describes a total of 10 RNA‐seq libraries from three species used in the comparative transcriptome analysis section.

**TABLE 1:** Details of the RNA‐seq samples in this study.

Scientific name	Sample ID	Organ	Sex	Read length (bp)	Layout	Total clean reads	Alignment rates (%)	SRA accessions
*Ailuropoda melanoleuca*	aml_PP	Liver	Male	150	Paired	21,302,638	96.67	SRR11301096
*Ailuropoda melanoleuca*	aml_YS	Liver	Male	150	Paired	22,807,879	94.02	SRR11301095
*Ailuropoda melanoleuca*	aml_CC	Liver	Male	150	Paired	20,345,289	95.98	SRR11301092
*Ailuropoda melanoleuca*	aml_HT	Liver	Female	150	Paired	32,302,526	94.97	SRR11301091
*Ailurus fulgens*	afu_1025	Liver	Female	150	Paired	31,498,532	88.91	SRR11301086
*Ailurus fulgens*	afu_0708	Liver	Female	150	Paired	23,780,054	89.54	SRR11301085
*Ailurus fulgens*	afu_0527	Liver	Female	150	Paired	24,855,251	90.56	SRR12158773
*Ailurus fulgens*	afu_0601	Liver	Male	150	Paired	18,490,226	86.64	SRR12158772
*Ursus maritimus*	umr_889A	Liver	Male	100	Paired	63,027,268	89.72	SRR20746746
*Ursus maritimus*	umr_891A	Liver	Male	100	Paired	61,427,888	90.50	SRR20746745

Splice sequences and low‐quality reads with quality scores below 20 were removed from the raw data using NGS QC Toolkit v2.3.3 (Patel & Jain, 2012) and assessed by FastQC v0.11.9 (http://www.bioinformatics.babraham.ac.uk/projects/fastqc/) to assess the quality of the raw sequencing data. Reads from each sample were mapped to the reference genome of the corresponding species using HISAT2 (Kim et al., 2019). The giant panda genome was downloaded from NCBI with version number ASM200744v2. The polar bear genome was downloaded from NCBI with version number UrsMar_1.0. The red panda genome was downloaded from DNA ZOO (https://www.dnazoo.org/). The SAM files were converted to BAM files using Samtools (Li et al., 2009). Finally, the expression abundance of all genes was estimated by featureCounts in the Subread package v1.6.4 (Liao et al., 2014).

To compare gene expression patterns among giant pandas, red pandas, and polar bears, we firstly identified 1:1 single‐copy orthologue genes in these species by using Orthofinder 2.3.7 (Emms & Kelly, 2015). Then, we normalized the expression levels of these orthologue genes with GeTMM (Gene Length Corrected TMM) to eliminate the effects of sequencing depth and gene length differences between species (Smid et al., 2018). The GeTMM normalization process was as follows: we first constructed an expression matrix for the samples, positioning the expression of orthologue genes as rows and sample names as columns. To correct for transcript length, we calculated a correction factor for each gene based on its length, thereby normalizing the sequencing depth of the samples. Subsequently, we filtered the normalized expression data by removing genes within the same species that exhibited zero expression across all samples.

Principal component analysis (PCA) for each sample was conducted using the prcomp function from the R package stats (R Core Team., 2013), with visualization through the ggplot function in the R package ggplot2 (Valero‐Mora, 2010). Spearman's rank correlation for clustering among samples was executed using the stats package's cor function, with heatmaps generated via the heatmap.2 function in the R package gplots. To further ensure the differences in sequencing depth and read length did not influence the clustering patterns observed, additional analyses including downsampling and read‐trimming were performed. Polar bear RNA‐seq data were downsampled to match the sequencing depth of the giant panda and red panda samples (approximately 20 million reads). Concurrently, read‐trimming was conducted for giant panda and red panda samples to adjust the read length to that of the polar bear samples (100 bp). Subsequent cluster analysis on these modified datasets confirmed their consistency with the clustering patterns of the unmodified datasets, as shown in Figure S1, underscoring the robustness of our sequencing strategy and analytical methodology.

Differential gene expression analysis was performed on the normalized GeTMM values using the R package edgeR (Robinson & Oshlack, 2010). Samples of liver tissue from giant and red pandas were compared to those from polar bears separately to determine fold changes of differential expression and *p*‐value after significance testing. Genes with a Benjamini–Hochberg false discovery rate ≤0.05 and |log_2_FC| ≥ 1 were screened for significantly differentially expressed genes (DEGs).

### 2.3 DNA library preparation, bisulphite sequencing, and data processing

Genomic DNA was extracted from liver tissues involved in whole genome bisulphite sequencing (WGBS) by using the Qiagen DNeasy Blood & Tissue kit. The extent of DNA degradation was then examined by Gelose Gel Electrophoresis to determine the presence of contamination. The OD260/280 ratio of 1 μL of uncontaminated DNA was assayed and then the concentration of DNA was accurately quantified using Qubit. After adding lambda DNA to the quantified genomic DNA, the DNA was fragmented with Covaris S220, interrupting it to a 200–300 bp fragment. The sequence was subsequently terminal repaired. The barcode sequences were ligated to the DNA fragments after methylation at the C site according to the instructions. These DNA fragments then underwent bisulphite conversion using EZ DNA Methylation‐GoldTM Kit (Zymo Research). The library concentration was quantified using Qubit and the size of the inserted sequence fragments was measured using the Agilent Bioanalyzer 2100 system. The prepared libraries were sequenced on the Illumina Hiseq X‐ten platform at Novogene (Beijing, China), generating 150 bp paired‐end reads. All reads obtained have been submitted to NCBI Sequence Read Archive with BioProject number PRJNA865071. Table 2 describes a total of 12 WGBS libraries from three species used in the comparative methylome analysis section.

**TABLE 2:** Details of the WGBS samples in this study.

Scientific name	Sample ID	Organs	Sex	Clean Base (G)	Alignment rates (%)	SRA accessions
*Ailuropoda melanoleuca*	aml_HT	Liver	Female	125G	65.2	SRR13334619
*Ailuropoda melanoleuca*	aml_CC	Liver	Male	138G	75.7	SRR13334621
*Ailuropoda melanoleuca*	aml_PP	Liver	Male	118G	76.7	SRR13334611
*Ailuropoda melanoleuca*	aml_YS	Liver	Male	131G	70.9	SRR13334610
*Ailuropoda melanoleuca*	aml_DN	Liver	Male	129G	77.4	SRR13286861
*Ailurus fulgens*	afu_1219	Liver	Female	136G	76.6	SRR18502787
*Ailurus fulgens*	afu_0527	Liver	Female	123G	77.7	SRR18502782
*Ailurus fulgens*	afu_0601	Liver	Male	119G	70.4	SRR18502788
*Ailurus fulgens*	afu_0429	Liver	Male	126G	73.4	SRR18502783
*Ursus maritimus*	umr_53412B	Liver	Male	111G	77.4	SRR20746744
*Ursus maritimus*	umr_53424A	Liver	Male	116G	77.4	SRR20746743
*Ursus maritimus*	umr_57208A	Liver	Male	92G	68.2	SRR20746742

Quality control of the raw data was performed by using Trim Galore v0.6.1 (https://github.com/FelixKrueger/TrimGalore) to remove adapter sequences and low‐quality reads with quality scores below 20, and remove sequences <20 bp in length. The filtration was then quantified and visualized by FastQC (http://www.bioinformatics.babraham.ac.uk/projects/fastqc/). The clean reads from liver samples of giant pandas, red pandas, and polar bears were transformed using Bismark_v0.20.0 (Krueger & Andrews, 2011) for G‐to‐A and C‐to‐T, respectively, and then aligned to their genomes by using the default minimum alignment score function, sorted with Samtools (Li et al., 2009). Duplicate reads were removed using deduplicate_bismark script in Bismark_v0.20.0. Bismark_methylation_extractor script in Bismark_v0.20.0 was used to extract numbers of converted and unconverted cytosines covering each locus. In this study, all samples were mapped to the corresponding genomes with alignment rates above 65%, which could be used for subsequent analysis (Table 2).

To investigate the genome methylation landscape, we first removed cytosine sites with less than 10× coverage, and then calculated the number of cytosines at CG, CHG, and CHH sites, and assessed the methylation level of each cytosines site (CG as CpG, CHG, and CHH). The methylation level of a cytosine site was counted as follows: Methylation level of cytosine site = mC/(mC + C), mC represents the number of methylation reads and C represents the number of unmethylation reads. We divided the sequence into 10 kb/bin and calculated the methylation level for each bin. The methylation level of each bin was counted as follows: Methylation level of bin = ΣmC/Σ(mC + C). The Violin plot for the overall distribution of methylation levels of each sample was performed on all bins of each sample. We extracted annotation files from GTF files, and defined gene functional regions using ‘genes’, ‘transcripts’, ‘promoters’, ‘intronsByTranscript’ and ‘exonsBy’ function in the R package GenomicFeatures. We then investigated the methylation level of defined gene functional regions, including promoter (upper 1000 bp of the transcript start sites), gene body, exon, and intron. Gene regions with upstream 2000 bp and downstream 2000 bp regions were divided into 20 bins, and then the average methylation levels of each bin were calculated by Python.

For PCA and Spearman's correlation distance clustering analysis, we focused on methylation levels of all comparable CpG sites across species. We constructed chain files for giant pandas against polar bears and red pandas against polar bears respectively using LAST v1080 (Hamada et al., 2017; Kiełbasa et al., 2011) and mapped individual CpG site of polar bear to the coordinates of giant panda and red panda genomes respectively using UCSC liftover (http://hgdownload.cse.ucsc.edu/admin/exe/linux.x86_64/liftOver) based on chain files. Subsequently, we extracted and combined methylation levels at compared CpG sites across all samples in different species. With the combined methylation levels, we then performed principal component analysis and Spearman correlation distance cluster analysis.

### 2.4 Differentially methylated genes (DMGs) identification

Differentially methylated regions (DMRs) refer to regions in the genome with different methylation levels between different groups of samples. We used DSS (Wu et al., 2015) based on methylation levels at compared CpG sites to identify the DMRs between the giant panda samples compared to the polar bear samples and the red panda samples compared to the polar bear samples. For DMR analysis, we set the window size of 500 bp, p.threshold of 1.00E‐04, and required each DMR to contain at least three CpG sites with methylation level differences ≥0.1. Gene‐body DMGs were identified only if the DMR overlapped with the gene‐body region by more than half of the DMR. To identify differentially methylated promoters between different groups, we calculate promoter methylation levels for all orthologue genes in the giant panda, red panda, and polar bear samples. The methylation level of 1000 bp promoter was calculated using the formula: Methylation level of promoter = ΣmC/Σ(mC+C), mC represents the number of methylation reads in promoter and C represents the number of unmethylation reads. The giant panda samples and red panda samples were compared with polar bear samples one by one to identify methylation differences at the promoter level of orthologue genes. Differentially methylated promoters are required to contain at least two CpG sites which were covered by more than 10 reads, the difference in methylation level between two groups was greater than two‐fold, and achieve a *p*‐value < 0.05 on a one‐tailed Wilcoxon rank‐sum test. Genes with differential methylation levels in the promoter region were defined as promoter DMGs.

### 2.5 Correlation analysis of DMGs and gene expression

The promoter and gene‐body regions have drawn a great deal of attention in studies of DNA methylation levels affecting gene expression (Elhamamsy, 2016), this study also explores these relationships. We analysed the correlations between these methylations and gene expressions. Firstly, we extracted both promoter DMGs and gene‐body DMGs whose absolute fold change (FC) in methylation level was greater than 2. We then extracted the expression level FC corresponding to these DMGs and excluded genes with |log_2_FC| ≤ 0.5. Finally, after log_2_ transformation of methylation level and expression level FCs, we calculated Spearman correlations between gene expression and methylation in promoter and gene‐body regions separately. A two‐tailed t‐test was used to assess the statistical significance of the correlations.

### 2.6 GO and KEGG enrichment analysis

The GO terms were extracted from EggNOGv5.0 (http://eggnog5.embl.de/#/app/home), and the KEGG pathways were extracted from the KEGG PATHWAY database (https://www.kegg.jp/kegg/pathway.html). The annotation information was imported into R and enrichment analysis was performed using the ‘enricher’ function of the ‘clusterProfiler’ package (Yu, 2018). A *p*‐value < 0.05 was considered to be significantly enriched.

### 2.7 Protein–protein interaction network analysis

To investigate the correlation between metabolism‐related DEGs, we used the STRING web server (https://string‐db.org) (version 11.0) to perform protein–protein interaction (PPI) network analysis. Network maps were drawn by Cytoscape v3.7.1 (Shannon et al., 2003). The intersection of genes (degrees) was calculated and the gene with the highest degree was considered the hub gene.

## 3 RESULTS

### 3.1 Overview of liver transcriptomes of giant pandas, red pandas and polar bears

**FIGURE 1:**
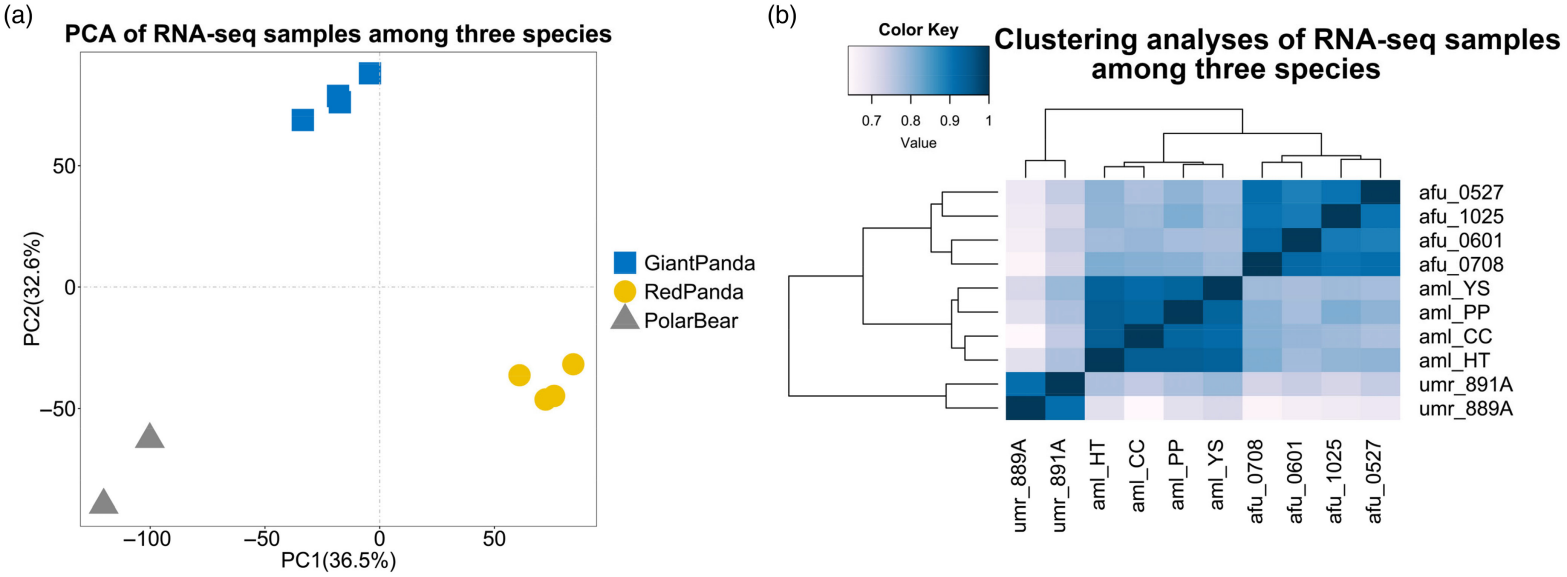
PCA and clustering analyses of the mRNA expressions for all samples. (a) PCA of the log‐transformed normalized expression levels of all orthologue genes across liver samples from different species. Species are represented by point shapes and colours. (b) Clustering analyses of the log‐transformed normalized expression levels of all orthologue genes across liver samples from different species. Distance between samples is measured by Spearman's rank correlation coefficient. Aml, giant panda; Afu, red panda; Umr, polar bear.

In this study, the reads alignment rates of RNA‐seq sequencing samples were 86.64%–96.67% (Table 1). In order to compare the homogeneity of gene expression before and after normalization, the coefficient of variation of the data was calculated, which allowed the dispersion of the two groups of data to be statistically measured. The coefficients of variation of expression after normalization were lower than the original expression, indicating that the normalization corrected for bias between species and among individual replicates (see Figure S2). We identified a total of 12,259 1:1 single‐copy orthologue genes in giant pandas, polar bears and red pandas. We performed PCA and Spearman's correlation distance clustering analysis on the expression of each sample (Figure 1a,b). The PCA analysis revealed that the transcripts of liver samples from the same species clustered well together, while that of liver samples from different species showed clear separation. Spearman's correlation distance clustering revealed that giant pandas clustered first with red pandas and did not form a sister group with the more closely related polar bears. This pattern may be related to their specific bamboo‐eating characteristics, suggesting that liver expressions of giant panda and red panda livers have evolved convergently driven by their diets.

### 3.2 Identification of DEGs

We compared RNA‐seq data from giant and red pandas to polar bears separately to identify DEGs (see Figure S3a,b). A total of 3687 DEGs were identified in giant pandas compared with polar bears, including 1700 upregulated and 1987 downregulated DEGs. In red pandas compared with polar bears, a number of 3926 DEGs were identified, including 1915 upregulated and 2011 downregulated DEGs.

### 3.3 Enrichment analysis of DEGs

We performed GO and KEGG enrichment analyses of DEGs to further understand their biological functions. We found that a large number of items related to immune function were enriched in upregulated genes in giant pandas compared to polar bears (Figure S4a–d and Tables S1–S4). The liver contains many immune‐associated cells, which play an important role in body's immunity (Kubes & Jenne, 2018). Additionally, upregulated DEGs in giant pandas were significant enrichment for lipid synthesis related entries, including cholesterol biosynthesis (GO:0006695), unsaturated fatty acid biosynthesis (GO:0006636), in addition to carbohydrate metabolism (GO:0005975) and arginine metabolic process (GO:0006525) (Table S30). Similarly, downregulated DEGs in giant pandas were significantly enriched for a large number of metabolism‐related entries.

Like in giant pandas, DEGs upregulated in red pandas compared to polar bears were also significantly enriched for immune‐related entries (Figure S4e–h and Tables S5–S8). Furthermore, both up‐ and downregulated DEGs in red pandas were enriched for a large number of nutrient metabolism‐related entries. The upregulated DEGs were predominantly anabolic, such as cellular carbohydrate biosynthesis (GO:0034637) and cholesterol biosynthesis (GO:0006695), and downregulated DEGs were predominantly catabolic, such as fat digestion and absorption (map04975) and lysine degradation (map00310) (Table S30).

### 3.4 Identification of metabolism‐related DEGs

Based on the enrichment results of DEGs, we extracted genes related to carbohydrate metabolism and energy production, lipid metabolism, amino acid and protein metabolism. A total of 642 DEGs were involved in processes related to nutrient metabolism in giant pandas compared with polar bears (Table S9), and protein–protein interaction network analysis of these genes revealed *EHHADH* and *STAT3* as core genes (see Figure S5). A total of 554 DEGs were involved in processes related to nutrient metabolism in red pandas compared to polar bears (Table S9), of which *GRHPR* and *HADH* were core genes (see Figure S6). *EHHADH* and *HADH* perform key roles in fatty acid oxidation (Ranea‐Robles et al., 2021; Wang et al., 2022), indicating that giant pandas and red pandas differ significantly in their fatty acid oxidizing capacity from polar bears. Furthermore, the roles of *STAT3* in mediating cellular responses to external signals and *GRHPR* in glyoxylate detoxification and metabolism suggest complex regulatory mechanisms underlying dietary adaptations (Dindo et al., 2019). These findings propose that the adaptation to a bamboo diet in giant pandas and red pandas involve not only shifts in gene expression related to nutrient metabolism but also intricate protein–protein interactions that govern these metabolic pathways.

**FIGURE 2:**
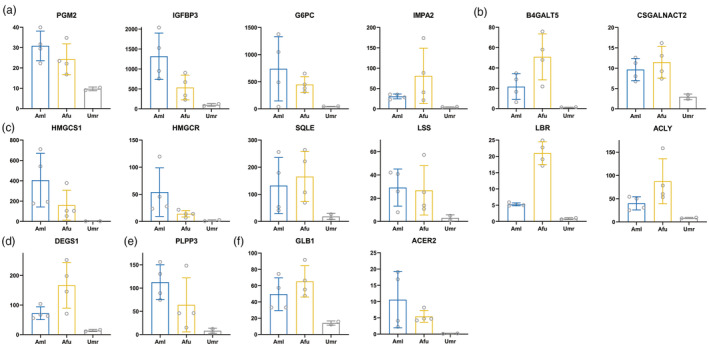
Scatter bar chart of upregulated DEGs that are convergently expressed in Aml versus Umr group and Afu versus Umr group. (a) Glucose catabolism‐related genes. (b) Other saccharide metabolism‐related genes. (c) Cholesterol synthesis‐related genes. (d) Unsaturated fatty acid synthesis‐related genes. (e) Phospholipid synthesis related genes. (f) Sphingolipid metabolism‐related genes. *X*‐axis indicates different species and *Y*‐axis indicates count‐per‐million (CPM) normalized expression. Aml, giant panda; Afu, red panda; Umr, polar bear.

**FIGURE 3:**
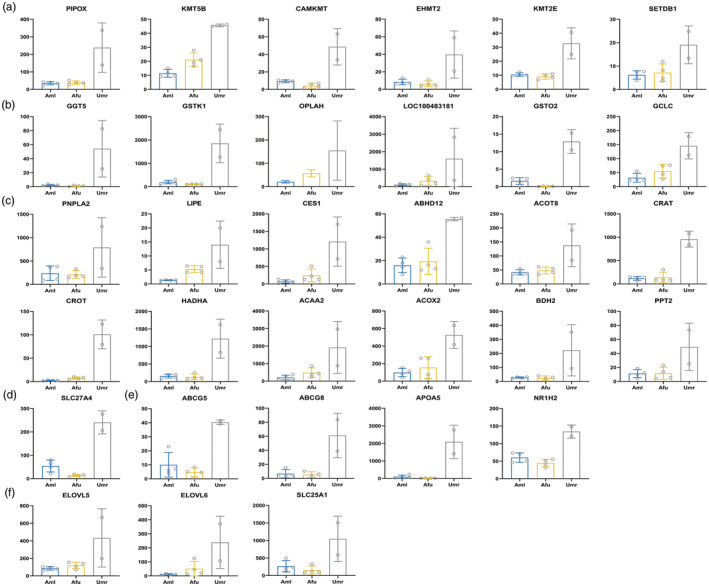
Scatter bar chart of downregulated DEGs that are convergently expressed in Aml versus Umr group and Afu versus Umr group. (a) Lysine degradation‐related genes. (b) Glutathione metabolism‐related genes. (c) Lipid catabolism‐related genes. (d) Fat digestion and absorption‐related genes. (e) Cholesterol clearance‐related genes. (f) Fatty acid synthesis‐related genes. *X*‐axis indicates different species and *Y*‐axis indicates CPM normalized expression. Aml, giant panda; Afu, red panda; Umr, polar bear.

We found that many DEGs related to nutrient metabolism showed similar trends of being up‐/downregulated in giant pandas and red pandas compared to polar bears (Figures 2 and 3). We defined these genes as convergently expressed genes. Among them, we identified 16 upregulated DEGs involved in metabolic processes such as glucose catabolism, polysaccharide synthesis, and the synthesis of unsaturated fatty acids, cholesterol, phospholipids, and sphingolipids. Additionally, we found 33 DEGs with downregulated convergent expression, mainly related to fat digestion and absorption, lipid catabolism, fatty acid synthesis, cholesterol clearance, as well as lysine degradation and glutathione metabolism. In addition, we compared the absolute expression of DEGs in giant panda, red panda, and polar bear. We found that most of the DEGs with downregulated convergent expression related to fat digestion and absorption, and lipid catabolism metabolism in both giant and red pandas had very low expression, and much lower than that in polar bears. This indicates that their low expression is a response to the low‐fat content of bamboo, rather than merely being lower than those in polar bears. These DEGs highlight the parallel metabolic adaptations to a bamboo diet in the two species.

### 3.5 DNA methylation profiles and patterns of the liver genomes of giant pandas, red pandas, and polar bears

The sequencing quality and genomic coverage of DNA methylation sequencing samples in this study are shown in Table 2. The sequence alignment rates of all samples were higher than 65%, which were similar to a previous study (Ren et al., 2019). We counted the number of cytosine sites in each sample and found that sites with >1 reads of cytosine site coverage in the sample accounted for more than 93% of all cytosine sites, and the sites with >5 reads of cytosine site coverage accounted for more than 76% of all cytosine sites (Table S10), which was higher than previous studies (Chen et al., 2018; Lister et al., 2008).

**FIGURE 4:**
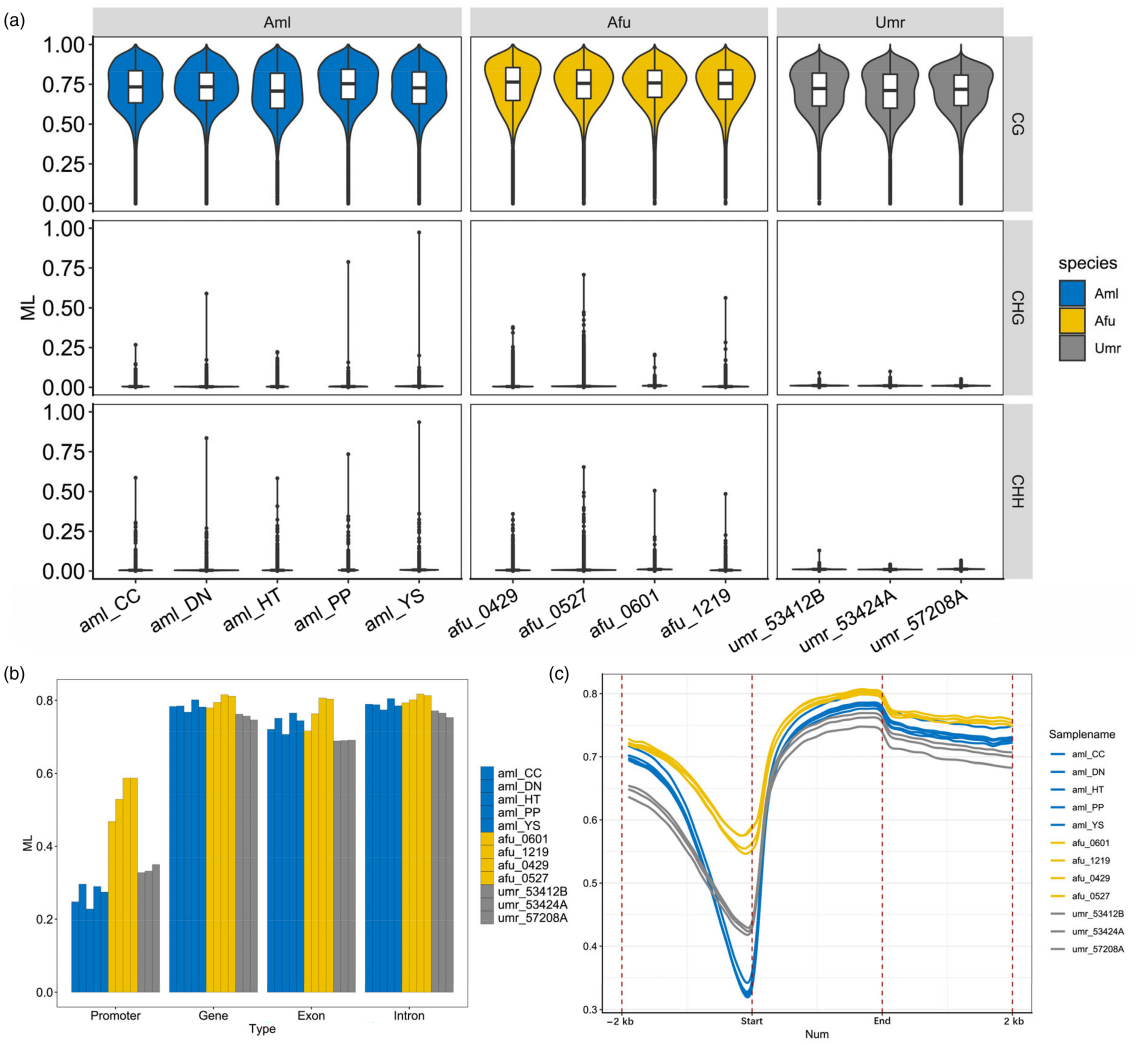
The DNA methylation levels and patterns. (a) Distribution of CG methylation levels across the genome for liver samples in giant pandas, red pandas, and polar bears. *X*‐axis indicates different sample among three species, *Y*‐axis indicates methylation levels. Every 10 kb is taken as a bin. The width of each violin indicates how many bins are below this methylation level. (b) The mean CG methylation level of different gene elements in liver samples among giant pandas, red pandas, and polar bears. *X*‐axis indicates different functional regions of genes, *Y*‐axis indicates methylation levels, different colours represent different species. (c) The dynamic changes of methylation levels in the upstream and downstream regions of genes in liver samples in giant pandas, red pandas and polar bears. *X*‐axis indicates different regions, *Y*‐axis indicates methylation level, Colours represent different samples. Aml, giant panda; Afu, red panda; Umr, polar bear.

Consistent with other mammals, the genomes of giant pandas, red pandas, and polar bears exhibit methylation in three cytosine sequence contexts: CG, CHG, and CHH. We divided the genome into 10,000 bp/bin and calculated the methylation levels of each bin at different cytosine site sequence contexts in the samples of giant pandas, red pandas, and polar bears (Figure 4a). The results showed that methylation levels of cytosine sites were similar among giant pandas, red pandas, and polar bears, and the methylation levels of CG‐type sites were significantly higher than those of CHG and CHH type sites in giant pandas, red pandas, and polar bears, with most of the regions having methylation levels above 0.5. This suggests that genomic methylation in the livers of giant panda, red pandas, and polar bears occurs mainly at the CpG site (Zhang et al., 2017).

Methylation levels at different sites in the genome have different functions. Since methylation primarily occurs at CG‐type motifs, we calculated average methylation levels of different regions of genes (promoter region, gene region, exon region, and intron region) at the whole genome level based on the CpG site type. The results showed that the average methylation levels in the gene, exon and intron regions were similar in giant pandas, red pandas, and polar bears, while the average methylation levels in the promoter region were distinct. The average methylation level in the promoter region of polar bears was slightly higher than that of giant pandas, while the average methylation level in the promoter region of red pandas was much higher than that of polar bears and giant pandas. In addition, the average methylation level of promoter region was the lowest, while the methylation levels of gene region, exon region, and intron region were higher in all three species (Figure 4b). To show the trend of methylation levels more visually, we divided the gene region and its adjacent upstream and downstream regions into 20 bins and made a trend graph based on the methylation levels of each bin (Figure 4c). The results showed that the trends of methylation levels in three species were similar. Methylation levels were gradually decreasing and then slightly increasing in the upstream 2 kb region of the gene‐body, and methylation levels in the gene region reached the highest, while methylation levels in the downstream 2 kb region of the gene‐body decreased slightly remaining at the same level. This is consistent with the results found in other animal studies (Li et al., 2018; Sevane et al., 2019). Interestingly, the methylation levels within 1000 bp upstream of the transcription start site in red pandas are much higher than in giant pandas and polar bears, which is consistent with the results of the average methylation levels in the promoter regions of the three species.

**FIGURE 5:**
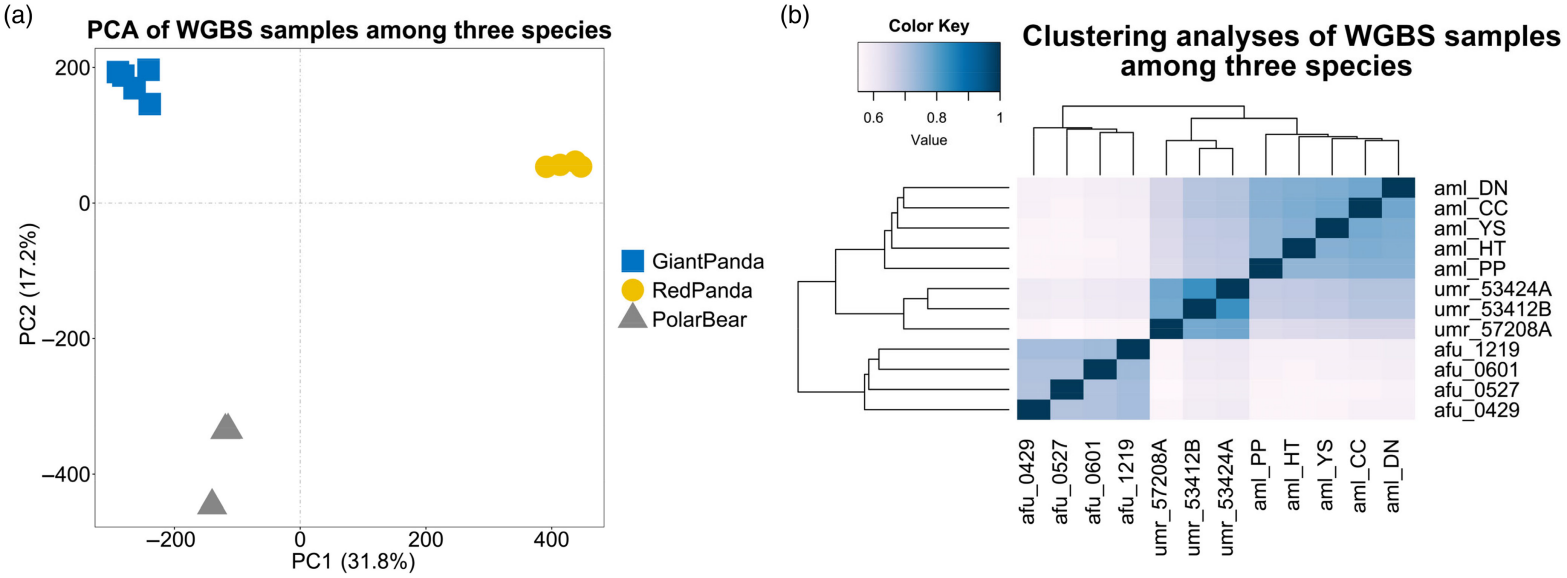
PCA and clustering analyses of the CG methylation levels for all samples. (a) PCA analyses of the CG methylation levels for liver samples from different species. Species are represented by point shapes and colours. (b) Clustering of liver samples based on CG methylation levels. Distance between samples is measured by Spearman's rank correlation coefficient. Aml, giant panda; Afu, red panda; Umr, polar bear.

To assess the rationality of sample grouping, we performed PCA and Spearman correlation distance clustering analysis on the samples (Figure 5a,b). Consistent with the transcriptome PCA results, the results of methylation PCA analysis showed that liver samples between different species completely separated at the PC1 level. However, contrary to the transcriptome clustering results, spearman correlation distance clustering analysis of methylation showed that giant pandas and the more closely related polar bears clustered into one group first, and red pandas clustered into one group alone. These observations indicate that the dietary adaptations influencing the transcriptomes might induce targeted methylation modifications in genes directly involved with processing unique dietary components, such as those associated with digestive enzymes, nutrient absorption, and metabolic pathways specific to compounds found in bamboo. However, these localized methylation changes do not sufficiently alter the overall methylome clustering pattern, which still exhibits phylogenetic lineage patterns. This phenomenon highlights the dual nature of epigenetic modifications like DNA methylation, serving both as a rapid response mechanism to environmental changes, such as diet, and a more stable genomic feature reflecting evolutionary trajectories.

### 3.6 Identification of gene‐body DMGs

We compared giant and red pandas with polar bears, respectively, to identify DMRs. A total of 38,742 DMRs (25,837 hypermethylated and 12,905 hypomethylated) were identified in giant pandas compared to polar bears, and a total of 50,195 DMRs (37,618 hypermethylated and 12,577 hypomethylated) were identified in red pandas compared to polar bears (Tables S11 and S12). Overall, the number of hypermethylated DMRs was higher than the number of hypomethylated DMRs in both comparisons. We performed densitometric statistics on the length of DMR regions in the different comparisons, and the length of DMRs ranged from 51 bp to over 2500 bp, with most DMRs concentrated in the range of 50–350 bp (Figure S7a,b).

DMRs localized in gene‐body region often play important functions in biological processes (Arechederra et al., 2018; Yi, 2017). In comparison of different species, 6726 (4757 hypermethylated and 1969 hypomethylated) and 5168 (3735 hypermethylated and 1433 hypomethylated) DMRs were found to be localized in gene‐body region of giant pandas compared to polar bears and red pandas compared to polar bears, respectively (see Figure S8a). Some of these genes had both hypermethylated DMRs and hypomethylated DMRs, reflecting the complexity of methylation distribution. Therefore, we performed enrichment analyses only for gene‐body DMGs with only hypermethylated DMRs or hypomethylated DMRs.

Both hypermethylated and hypomethylated gene‐body DMGs of giant pandas were significantly enriched for a large number of nutrient metabolism‐related entries compared to polar bears (Figure S9a–d, Tables S13–S16 and S31). For example, lipid oxidation (GO:0034440) and tryptophan metabolism (map00380) were significantly enriched in hypermethylated genes, and steroid biosynthesis (map00100) and α‐amino acid metabolism (GO:1901605) were significantly enriched in hypomethylated genes. Moreover, hypermethylated genes were significantly enriched for entries related to organic acid metabolism and transport and peroxisomal‐related entries.

Similar to giant pandas, red pandas were also significantly enriched for a large number of nutrient metabolism‐related entries on both hypermethylated and hypomethylated gene‐body DMGs compared to polar bears (Figure S9e–h, Tables S13–S16 and S31). For example, the hypermethylated genes were significantly enriched for catabolic‐related entries such as pyruvate metabolism (ko00620), triglyceride lipase activity (GO:0004806), endopeptidase activity (GO:0004175), and peroxisome (ko04146). Hypomethylated genes, on the other hand, were significantly enriched for entries on fatty acid degradation (ko00071), cholesterol biosynthesis (GO:0006695), and lysine degradation (ko00310).

### 3.7 Identification of promoter DMGs and analysis of DNA methylation regulation

Studies have shown that promoter region of gene is the main functional region that regulates gene expression, and methylation in promoter region often results in silencing of gene expression (Moore et al., 2013). To explore the correlation between dietary changes and regulation of gene expression, we investigated the differences in methylation levels in the promoter region of each gene. In the comparison of different species, 4703 promoter DMGs were identified in giant pandas compared to polar bears, including 2132 hypermethylated and 2571 hypomethylated genes (see Figure S8b). In the comparison between red pandas and polar bears, we identified 4473 promoter DMGs, of which 3225 genes were hypermethylated and 1248 genes were hypomethylated (see Figure S8b).

To clarify the relationship between methylation status and expression of genes, we integrated and analysed genes with differential methylation in promoter or gene‐body regions with transcriptome data, and calculated Spearman's correlation coefficients (see Figure S10a,b). We found a significant negative correlation between methylation levels and gene expression (promoter region: *p* = 0.022; gene‐body region: *p* < 0.0001). However, it is well‐established that promoter region methylation typically leads to gene silencing, while the functional implication of gene‐body methylation remains unclear. Therefore, we extracted genes that were negatively correlated with promoter region methylation and gene expression for subsequent analysis. In giant pandas, 805 genes negatively correlated between promoter region methylation and gene expression compared with polar bears. Of these, 415 were hypomethylated and highly expressed and 390 were hypermethylated and lowly expressed. In red pandas, 820 genes were negatively correlated between promoter region methylation and gene expression compared with polar bears, of which 252 were hypomethylated and highly expressed and 568 were hypermethylated and lowly expressed.

**FIGURE 6:**
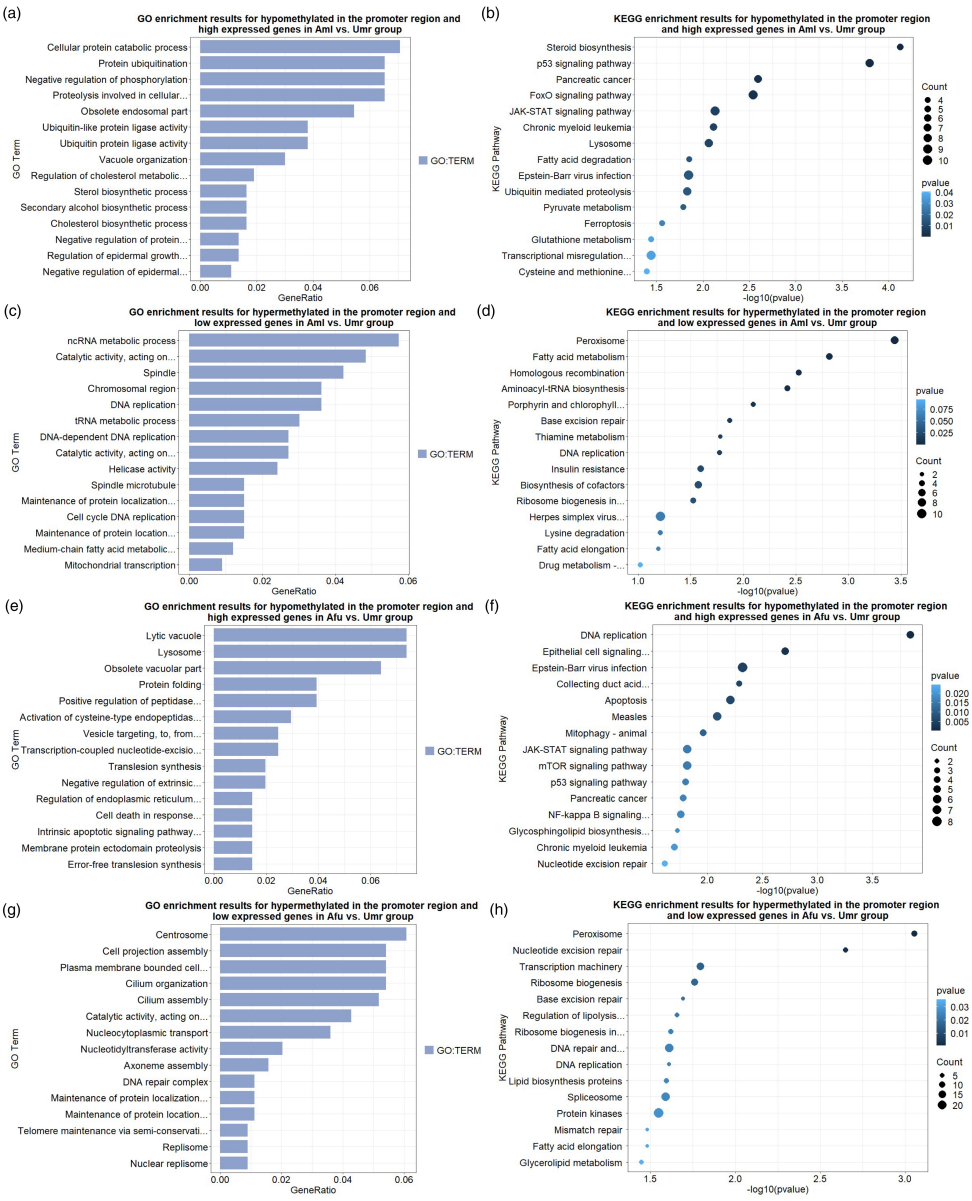
Significantly enriched GO categories and KEGG categories of genes with promoter region methylation levels negatively correlated with expression. (a) Significantly enriched GO categories for genes with higher expression and hypomethylated promoter in Aml versus Umr group. (b) Significantly enriched KEGG categories for genes with higher expression and hypomethylated promoter in Aml versus Umr group. (c) Significantly enriched GO categories for genes with lower expression and hypermethylated promoter in Aml versus Umr group. (d) Significantly enriched KEGG categories for genes with lower expression and hypermethylated promoter in Aml versus Umr group. (e) Significantly enriched GO categories for genes with higher expression and hypomethylated promoter in Afu versus Umr group. (f) Significantly enriched KEGG categories for genes with higher expression and hypomethylated promoter in Afu versus Umr group. (g) Significantly enriched GO categories for genes with lower expression and hypermethylated promoter in Afu versus Umr group. (h) Significantly enriched KEGG categories for genes with lower expression and hypermethylated promoter in Afu versus Umr group. The top 15 most significantly enriched items/pathways are shown. *X*‐axis indicates the GeneRatio of the GO enriched items or the −log_10_ (*p* value) of KEGG enriched pathways, *Y*‐axis indicates the name of the item/pathway. The number of genes in the KEGG enriched pathway is indicated by the size of the circle. Aml, giant panda; Afu, red panda; Umr, polar bear.

Subsequently, we performed GO and KEGG enrichment analyses for the negatively correlated genes (Figure 6a–h). Hypermethylated and lowly expressed genes in giant pandas were significantly enriched for a large number of items related to catabolism, including fatty acid β‐oxidation (GO:0006635) and exopeptidase activity (GO:0008238) compared to polar bears (Tables S21–S24 and S32). Similarly, hypomethylated and highly expressed genes are also significantly enriched for a large number of items related to nutrient metabolism, including pyruvate metabolism (map00620) and cholesterol biosynthesis process (GO:0006695).

Similarly, the hypermethylated genes with lower expression and hypomethylated genes with higher expression of red pandas were both significantly enriched for a large number of items related to nutrient metabolism (Tables S25–S28 and S32). For example, fatty acid elongation (ko00062) and lysine degradation (ko00310) were significantly enriched in the hypermethylated genes with lower expression. And, sphingolipid biosynthesis process (GO:0006688), positive regulation of endopeptidase activity (GO:0010950) were significantly enriched in the hypomethylated genes with higher expression.

### 3.8 Identification of convergent methylated DEGs related to nutrient metabolism

**FIGURE 7:**
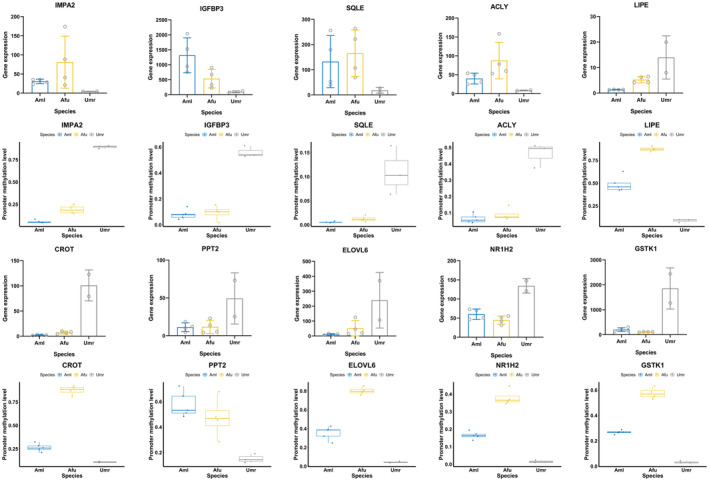
Scatter bar chart and box plot of genes negatively associated with convergent expression and convergent methylation in Aml versus Umr group and Afu versus Umr group. *X*‐axis indicates different species, *Y*‐axis in the scatter bar chart indicates CPM normalized expression, and *Y*‐axis in the box plot indicates gene promoter region methylation levels. Aml, giant panda; Afu, red panda; Umr, polar bear.

To clarify the regulation of DNA methylation of nutritional metabolism‐related DEGs in the three species, we extracted nutritional metabolism‐related DEGs that were negatively correlated with differential methylation levels in their promoter regions. We identified 283 genes between giant pandas and polar bears, and 259 genes between red pandas and polar bears (Table S29). Many of the nutrient metabolism‐related genes exhibited convergent hypomethylated with higher expression or hypermethylated with lower expression patterns in both two panda species compared to polar bears. Convergent hypomethylated and highly expressed genes include glucose catabolism‐related genes *IMPA2*, *IGFBP3*, and cholesterol synthesis‐related genes *SQLE*, *ACLY*. Convergent hypermethylated and lowly expressed genes include lipid metabolism and transport‐related genes *LIPE*, *CROT*, *PPT2*, *ELOVL6*, *NR1H2*, and glutathione metabolism‐related genes *GSTK1* (Figure 7).

## 4 DISCUSSION

The evolution of biological phenotypes can occur through a variety of mechanisms, and many adaptive phenotypic changes have been shown to be caused by changes in gene expression (Abzhanov et al., 2006; Carroll et al., 2013; Davidson, 2010; Manceau et al., 2011), while changes in DNA methylation are important epigenetic modification that affect gene expression. DNA methylation is a heritable covalent modification that is common in most species (Robertson, 2005; Sánchez‐Romero et al., 2015; Xu et al., 2020). It is widely accepted that transcription activation is negatively correlated with methylation of gene promoter region (Beletskaya et al., 2000; Christensen & Josephsen, 2004). An increasing number of studies have demonstrated that DNA methylation plays an important role in the adaptive evolution of species. For example, in the blind mole rat (*Spalax galili*), methylation of the promoter region of the P53 gene and then repression change mediates the adaptation to hypoxia, temperature, and pH changes (Zhao et al., 2016). Temperate lotus has higher global methylation levels compared to tropical lotus, which helps it form thicker rhizomes to adapt to cooler temperatures (Li et al., 2021). Consumption of a protein‐restricted diet in pregnant rats leads to hypomethylation of the GR gene promoter in their offspring, which promotes GR expression and thus enhances glucose metabolism (Lillycrop et al., 2007). Thus, by combining comparative transcriptome and comparative methylome studies, we are able to understand the molecular basis and methylation regulation of adaptation to high‐fibre, low‐fat bamboo diets in giant and red pandas.

The liver is an important organ for digestion and metabolism. Our research revealed that a significant number of genes associated with carbohydrate metabolism exhibit higher expression levels in giant pandas compared to polar bears (Table S30). These genes are predominantly engaged in various biological processes, including carbohydrate metabolism, glucose oxidation, glycolysis, as well as the synthesis and catabolism of polysaccharides, the hydrolysis of oligosaccharides, and inositol synthesis. This heightened expression of genes related to glycogen synthesis and glucose oxidation in giant pandas suggests a notable demand for saccharides, surpassing that of polar bears, alongside an enhanced capacity for glucose metabolism. This could be attributed to giant pandas' diet. Bamboo, which forms the bulk of their diet, is largely indigestible, with soluble saccharides and other accessible carbohydrates serving as the primary energy sources. Therefore, the high expression of genes involved in glucose metabolism potentially enables giant pandas to more efficiently utilize the saccharides present in bamboo, facilitating their adaptation to a diet predominantly composed of this challenging food source. Among the key genes related to carbohydrate metabolism, *IMPA2*, *IGFBP3*, *GLA*, *MANBA*, and *NEU1* exhibited a significant hypomethylation and higher expression pattern in giant pandas. *IMPA2* converts inositol monophosphate to free inositol through dephosphorylation, which is an important source of ATP synthesis in the organism (Bloch et al., 2010; Colazingari et al., 2014; Gardner et al., 2001). *IGFBP3* has a potential role in maintaining energy balance and regulating glucose metabolism (Kim, 2013). Both *IMPA2* and *IGFBP3* are important genes that mediate the oxidative capacity of glucose, suggesting that demethylation of carbohydrate metabolism‐related genes may be involved in the enhanced capacity of giant pandas to metabolize saccharides during the transition from a meat to a bamboo diet in order to adapt to a low‐fat bamboo diet. Previous studies on deep‐sea animals exposed to limited food supply show that genes involved in energy metabolism processes such as carbohydrate catabolism and cellular respiration were positive selected as an adaptation to the harsh environment of energy shortage in food (Kobayashi et al., 2012; Lan et al., 2017). Similar to this, the demethylation of genes related to oxidative capacity of carbohydrate in giant pandas in this study may also be an adaptive strategy for the efficient use of limited energy in bamboo. *GLA* and *MANBA* synergistically hydrolyse mannans to oligosaccharides or fermentable sugars, and *NEU1* is a sialidase that mainly catalyses the hydrolysis of oligosaccharides and glycoprotein terminal sialic acid residues (d'Azzo & Bonten, 2010; Moreira, 2008). Mannans are a major group of hemicellulose, and bamboo contains a large amount of hemicellulose, which is difficult for giant pandas to digest and utilize. The hypomethylation and higher expression of these genes related to polysaccharides or oligosaccharides hydrolysis facilitate the digestion and absorption of hemicellulose components in bamboo for giant pandas, which is also similar to the result of He et al. (2020).

Similarly, genes significantly overexpressed in red pandas compared to polar bears were found to be notably enriched on several metabolic pathways related to carbohydrate metabolism (Table S30). These pathways include the synthesis of carbohydrates, dephosphorylation of carbohydrates, metabolism of glucose, and metabolism of galactose. Among these genes, *PGM2*, *IGFBP3*, *G6PC*, *IMPA2*, *B4GALT5*, and *CSGALNACT2* were highly expressed in both red pandas and giant pandas compared to polar bears (Figure 2). For red pandas, the available carbohydrates in bamboo are also a major source of energy, suggesting that the high expression of genes for carbohydrate metabolism in red pandas is also a reflection of energy requirements. Notably, *IMPA2* and *IGFBP3* were also significantly hypomethylated in red pandas compared to polar bears (Figure 7). This suggests that red pandas show a similar trend of demethylation of key genes for carbohydrate metabolism during dietary changes as that of giant pandas. Interestingly, this pattern of high expression of genes related to carbohydrate uptake and utilization has also been found in comparisons of adult giant pandas and red pandas with suckling stages (Yizhen et al., 2023). Previous studies have shown that genes related to nutrient metabolism in the two panda species have evolved convergently at the genomic level (Hu et al., 2017). Therefore, the appearance of similar expression and methylation regulation patterns of some carbohydrate metabolism‐related genes in the two panda species suggests that convergent evolution of modification of DNA methylation patterns in some important genes in the two panda species has also occurred during the evolutionary process of diet shift.

The liver is an important site for the regulation of lipid synthesis and catabolism. We found that many significantly downregulated genes were associated with lipid metabolism and transport in giant pandas compared to polar bears (Table S30). Among these downregulated genes, the promoter regions of *ACAT2*, *LIPE*, *HSD11B1*, *CES1*, *NUDT19*, *PNPLA4*, *CROT*, *CPT2*, *ELOVL6*, *PPT2*, *OXSM*, *NR1H2*, and *IRAK1* showed significant hypermethylation, and they are crucial in lipid digestion and absorption, lipid catabolic processes, lipid acyl coenzyme A metabolic processes, fatty acid synthase activity, and regulation of cholesterol transport (Gao et al., 2019; Houten & Wanders, 2010; Kienesberger et al., 2009; Klucken et al., 2000; Ofman et al., 2006; Rana et al., 2016; Rudel et al., 2001; Schwartz et al., 2000; Shi et al., 2017; Soni et al., 2004; Soyombo & Hofmann, 1997; Wamil et al., 2011). For example, *ACAT2* is required for intestinal cholesterol absorption, *LIPE* catalyses the second step of lipolysis, *HSD11B1* increases lipoprotein lipase activity, and *CES1* mediates hydrolysis in adipose tissue (Rudel et al., 2001; Soni et al., 2004; Wamil et al., 2011). Hypermethylation of these genes in giant pandas results in low lipid metabolism, suggesting a methylation regulation to adapt to the shift in specific diets. Similarity, we also found that many low‐expressed genes related to lipid metabolism in red pandas compared to polar bears were convergent with those in giant pandas, such as the genes involved in fat digestion and absorption, lipid catabolism, cholesterol clearance (*SLC27A4*, *PNPLA2*, *LIPE*, and *NR1H2*) (Figure 3). Some key genes among the above convergent low‐expressed genes in giant and red pandas also show convergent evolutionary trends of promoter hypermethylation, such as *LIPE*, *CROT*, *PPT2*, *ELOVL6*, and *NR1H2*, suggesting that the expression of genes related to lipid metabolism in red pandas may also be regulated by methylation during the evolution of diet. Previous studies have also shown that a very low‐fat diet can induce a reduction in low‐density lipoprotein cholesterol, that bamboo contains only 0.3% fat and no cholesterol, and that hypermethylation and lower expression of genes related to lipid metabolism and transport in giant and red pandas may be related to long‐term bamboo‐eating adaptations (Bamboo shoots raw, 2019; Dreon et al., 2000). In contrast, polar bears predominantly eat a high‐lipid and high‐protein diet. Previous genomic studies have shown that several genes involved in lipid or fatty acid degradation are strongly selected in two carnivores, polar bear and cat, and may be associated with adaptation to a high‐fat diet, which is consistent with the results of the present study (Liu et al., 2014; Montague et al., 2014). Polar bears have very high cholesterol levels in plasma and high cholesterol is a major risk factor for the development of cardiovascular disease (Cannon et al., 2010; Ciesielski et al., 2018). Previous studies have identified nine fixed missense mutations in the *APOB* gene in polar bears compared to pandas that reduce the potential for cholesterol to enter into cells (Liu et al., 2014). The higher expression of genes related to cholesterol scavenging in polar bears in this study may also be able to counteract the negative effects of high fat intake in the diet.

Notably, we also found a large number of genes related to bioactive lipid synthesis and structural lipid metabolism showed high expression trends in giant pandas compared to polar bears (Table S30). They are mainly involved in biological processes such as cholesterol biosynthesis, steroid biosynthesis, terpenoid skeleton biosynthesis, glycerophospholipid metabolism, and sphingolipid metabolism (Burke & Huff, 2017; Nishi et al., 2000; Zerenturk et al., 2013). In addition, most of the above upregulated genes showed hypomethylation of the promoter region compared to polar bears, such as *HMGCS1*, *SQLE*, *SC5D*, *ACLY*, *DHCR24*, and *ACHE*. Cholesterol is an important component of biological membranes and a precursor to physiologically active substances such as bile acids and steroid hormones, and the organism usually obtains cholesterol from two pathways: dietary intake and de novo biosynthesis in liver (Simons & Vaz, 2004). Previous studies have demonstrated that when dietary cholesterol levels are high, cholesterol synthesis in liver is inhibited by a strong negative feedback mechanism (Goldstein & Brown, 1990); however, when dietary cholesterol is not available, such as in plant‐based diets, cholesterol synthesis in liver increases to meet the organism's demand for cholesterol (Jurevics et al., 2000).

This study found that genes involved in cholesterol synthesis in giant pandas show a pattern of hypomethylation and high expression, and that the increased expression of these genes is an adaptive compensatory strategy for giant pandas after their conversion to a specialized (cholesterol‐free) bamboo diet. During evolution, gene expression has been strongly shaped in an adaptive manner to diet, and *FADS1* and *FADS2* encode rate‐limiting enzymes in the synthesis of ω‐3 and ω‐6 long‐chain polyunsaturated fatty acids, which are rare in plant‐based diets but abundant in seafood (Glaser et al., 2010; Mathieson, 2020; Rees et al., 2020). Previous studies have shown that agricultural populations with predominantly plant‐based diets have upregulated *FADS1* expression, whereas the same genetic variation is rare in Greenlandic Inuit, who traditionally consume seafood (Harris et al., 2019; Mathieson & Mathieson, 2018). *FADS* genetic variation has been associated with metabolic and inflammatory responses, and enhanced *FADS1* synthesis in plant‐based populations has been interpreted as a result of positive selection of their diets as a means of reducing the likelihood of disease risk (Harris et al., 2019; Koletzko et al., 2019; Mathieson & Mathieson, 2018). Notably, our study presented similar results that *FADS1* exhibited a pattern of hypomethylation and high expression. The scarcity of unsaturated fatty acids in bamboo and enhanced *FADS1* expression induced by hypomethylation in the promoter region may also be the result of diet adaptation to reduce the adverse effects of the lack of such lipids by enhancing the synthesis of unsaturated fatty acids in giant pandas. *ACHE* encodes acetylcholinesterase, which rapidly hydrolyses acetylcholine, and *AGPAT1* is involved in the de novo biosynthesis of phospholipids (Tsim & Soreq, 2013; Yamashita et al., 1997). *NEU1* catalyses the desialylation of complex polysialo‐gangliosides to produce ganglioside *GM1* (Smutova et al., 2014; Timur et al., 2015).

Both phospholipids and sphingolipids are important components of biological membranes, and they are also involved in processes such as neuroprotection and signal transduction (Machala et al., 2019; Nadeau et al., 2019). The results of this study show that phospholipids and sphingolipids synthesis genes in giant pandas show a hypomethylation and high expression pattern, which may also reduce the negative effects of the lack of these lipids by enhancing the synthesis capacity. *DGAT2* is involved in catalysing the final and critical step in triglyceride biosynthesis (Cases et al., 1998). Previous studies have pointed out that Bornean orangutans (*Pongo pygmaeus*) living in a food‐deficit environment would be better at storing fat than Sumatran orangutans (*Pongo abelii*) with a stable food supply (Mattle‐Greminger et al., 2018). This metabolic change could provide a physiological buffer against starvation, and the trend of hypomethylation and high expression of the fat synthesis gene *DGAT2* in giant pandas in this study may reflect a physiological buffering adaptive response of giant pandas for adaption to a low‐energy bamboo diet (Knott, 1998). Compared with polar bears, we also found that most of the above genes in red pandas showed similar expression trends to those in giant pandas (Table S30). This involves genes in the cholesterol synthesis pathway (*HMGCS1*, *HMGCR*, *SQLE*, *LSS*, *LBR*, *ACLY*), unsaturated fatty acid synthesis (*DEGS1*), phospholipid synthesis (*PLPP3*), sphingolipid metabolism (*GLB1*, *ACER2*), which were significantly highly expressed in red pandas (Figure 2). Similarly, genes related to bioactive lipid synthesis and structural lipid metabolism were also significantly highly expressed in the livers of adult giant and red pandas compared to the suckling period, suggesting that the expression of genes related to digestive and metabolism have been evolving convergently in the two panda species during the dietary transition from juvenile to adult (Yizhen et al., 2023). Compared to polar bear, some key genes such as *SQLE* and *ACLY* were also significantly hypomethylation in red pandas in convergence with that in giant pandas (Figure 7), suggesting that the cholesterol synthesis requirement of red pandas is also increased after bamboo‐eating. The convergent hypomethylation levels of these genes indicate a convergent adaptation to bamboo diet in both giant and red pandas.

The liver is also an important site of amino acid metabolism. As an essential amino acid, lysine is crucial for mammalian growth, development, and nutritional metabolism (Tomé & Bos, 2007). Deficiency in lysine can result in diseases such as anaemia, impaired protein metabolism, and impaired fatty acid metabolism (Flanagan et al., 2010; Ghosh et al., 2010; Rushton, 2002). Unlike meat and certain green plants, bamboo contains very low levels of lysine (Bamboo shoots raw, 2019). Genes associated with lysine degradation (*AADAT*, *SETDB1*) showed a significant trend of hypermethylation and lower expression in giant pandas compared to polar bears. Similar to giant pandas, the expression of genes related to lysine degradation (*EZH1*, *KMT2E*) was also significantly hypermethylated in red pandas compared to polar bears. *AADAT* mediates the transamination reaction during lysine degradation, *SETDB1*, *EZH1*, and *KMT2E* regulate the methylation level of lysine residues during lysine degradation (Goh et al., 2002; Herz et al., 2013; Konze et al., 2013). In order to maintain the metabolic balance of lysine in body, the relatively low lysine degradation capacity to compensate for the lower lysine intake levels may explain the hypermethylation suppression of gene expression in lysine degradation‐related pathways in giant and red pandas. In addition, the suppression of gene expression related to lysine catabolism could reduce the cellular demand for lysine in vivo and improve the effectiveness of lysine utilization, helping giant and red pandas to survive in a low lysine diet (Cleveland et al., 2008).

Compared with polar bears, genes related to glutathione metabolism in giant pandas (*GSTK1*, *NAT8*) and red pandas (*GSTK1*, *GSTO1*, *OPLAH*) showed a significant trend of hypermethylation and lower expression (Figure 7). *GSTK1* and *GSTO1* are glutathione transferases that play an important role in inflammatory processes such as obesity, insulin resistance, and lipid peroxidation induced by high‐fat diets (Liu et al., 2012; Menon et al., 2017; Mosialou et al., 1993). *NAT8* and *OPLAH* are associated with the synthesis of glutathione, which has a role in attenuating lipid peroxidation responses and detoxification under high‐fat diets (Chen et al., 1998; Ketterer et al., 1983; Luo et al., 2021; Younes & Siegers, 1981). Compared to polar bears, we found that hypermethylated and lowly expressed genes in giant pandas were significantly enriched for the metabolism of xenobiotics by cytochrome P450 (*GSTK1*, *HSD11B1*), drug metabolism‐other enzymes (*CES1*, *UCK1*). These involved genes such as *HSD11B1* and *CES1* that are also related to lipid homeostatic regulation under high‐fat diet, and *UCK1* catalyses the phosphorylation of cytotoxic ribonucleoside analogues and reduces cytotoxicity (Soni et al., 2004; Van Kuilenburg & Meinsma, 2016; Wamil et al., 2011). Compared to polar bears, hypermethylated and lowly expressed genes were enriched for similar items in red pandas, including xenobiotic metabolism by cytochrome P450 (*GSTK1*, *GSTO1*) and drug metabolism‐cytochrome P450 (*GSTK1*, *GSTO1*). High purine and fat diets were known to induce gout and cardiovascular disease (Choi et al., 2004; Wang et al., 2009). Unlike meat, which is rich in purines, fats, and proteins, bamboo is predominantly fiber‐based, and genes associated with lipid homeostasis, resistance to lipid peroxidation, and detoxification are repressed by methylation in giant and red pandas, this is possibly also a reflection to match with bamboo diet during the evolution of diet. On the other hand, studies have shown that the addition of high animal food leaded to metabolic disorders in giant pandas, which showed loss of weight, frequent mucus excretion, lack of appetite, and reduced activity, potentially due to hypermethylation suppression of genes related to resistance to lipid peroxidation and detoxification in giant and red pandas (Knight et al., 1982).

The content of arginine in bamboo is much lower than that in animal meat (Bamboo shoots raw, 2019). We found that the expressions of genes related to arginine synthesis (*ASS1*, *DDAH1*) were upregulated in giant pandas by demethylation. *ASS1* and *DDAH1* encode argininosuccinate synthase and dimethylarginine dimethylaminohydrolase, respectively, which are key enzymes in arginine synthesis. Upregulation promoted by demethylation of *ASS1* and *DDAH1* in giant pandas could enhance arginine synthesis, thereby offsetting the limited supply of arginine in bamboo. Notably, *ASS1* and *DDAH1* also contribute to the regulation of vascular NO production to prevent inflammatory processes such as atherosclerosis (Goodwin et al., 2004; Liu et al., 2016). With long‐term consumption of plant foods reducing the incidence of cardiovascular disease, giant pandas dieted on high‐fiber, low‐fat bamboo and the aforementioned genes that reduce atherosclerosis show a hypomethylation and high expression pattern that seems to contradict their dietary habits (Kahleova et al., 2018). We noted that giant pandas have increased cholesterol synthesis and reduced cholesterol transport and clearance, which may contribute to cholesterol accumulation in blood vessels and consequently to vascular atherosclerosis. Previous studies have shown that vitamin B12 deficiency occurs in people on a long‐term vegetarian diet and that deficiency may counteract the benefits of a vegetarian diet in preventing cardiovascular disease (Pawlak, 2015). Despite being vegetarian, giant pandas are in fact at high risk of cardiovascular disease (Qiu & Mainka, 1993). So the upregulation of expression of the genes mentioned above may be an adaptive disease‐fighting strategy adopted by giant pandas in response to their bamboo diet.

## 5 CONCLUSION

In summary, genes involved in oxidative capacity of carbohydrate metabolism and hemicellulose hydrolysis in giant pandas are regulated by hypomethylation to better utilize carbohydrate in bamboo for energy supply. Further, genes involved in fat digestion and absorption, fatty acid metabolism, lysine degradation, resistance to lipid peroxidation, and detoxification are regulated by hypermethylation. While genes involved in bioactive lipid, structural lipid, and arginine synthesis and reduced atherogenesis are regulated by hypomethylation regulation. This is an adaptation strategy of giant pandas to the low‐fat diet and low‐amino acid of bamboo. These results indicate that DNA methylation might play an important role in adaptive regulation during the diet shift of giant pandas. Following the same diet shift, many genes in above metabolic processes show similar methylation and expression patterns in red pandas, which is also an adaptive result of red pandas in response to the diet shift. However, we also find that some of the diet‐related convergent expressed genes in giant and red pandas do not exhibit significant methylation regulation patterns. They may be genetically regulated by other aspects, such as histone modifications, non‐coding RNAs, or cytokines, and this part of the work needs to be further investigated.

## FUNDING INFORMATION

The research was funded by the National Natural Science Foundation of China (31770574) and the Natural Science Foundation of Sichuan Province (No. 2022NSFSC0126). Support was provided for polar bear sampling and the employees at Aarhus University under the AMAP CORE programmed by the DANCEA (Danish Cooperation for Environment in the Arctic).

## CONFLICT OF INTEREST STATEMENT

The authors declare that they have no conflict of interest.

## Supporting information


Figure S1.

Table S1.

## Data Availability

The high‐throughput sequencing data from this study have been submitted to the NCBI with the project accession PRJNA865071.
