# Supplementary material for: Comparative transcriptome and methylome of polar bears, giant and red pandas reveal diet‐driven adaptive evolution
Source: Evol Appl. 2024 Jun 17;17(6):e13731. doi: 10.1111/eva.13731 (PMC11183199; doi:10.1111/eva.13731)
Supplement: Supplementary file 1 — Figure S1. [file EVA-17-e13731-s001.docx]

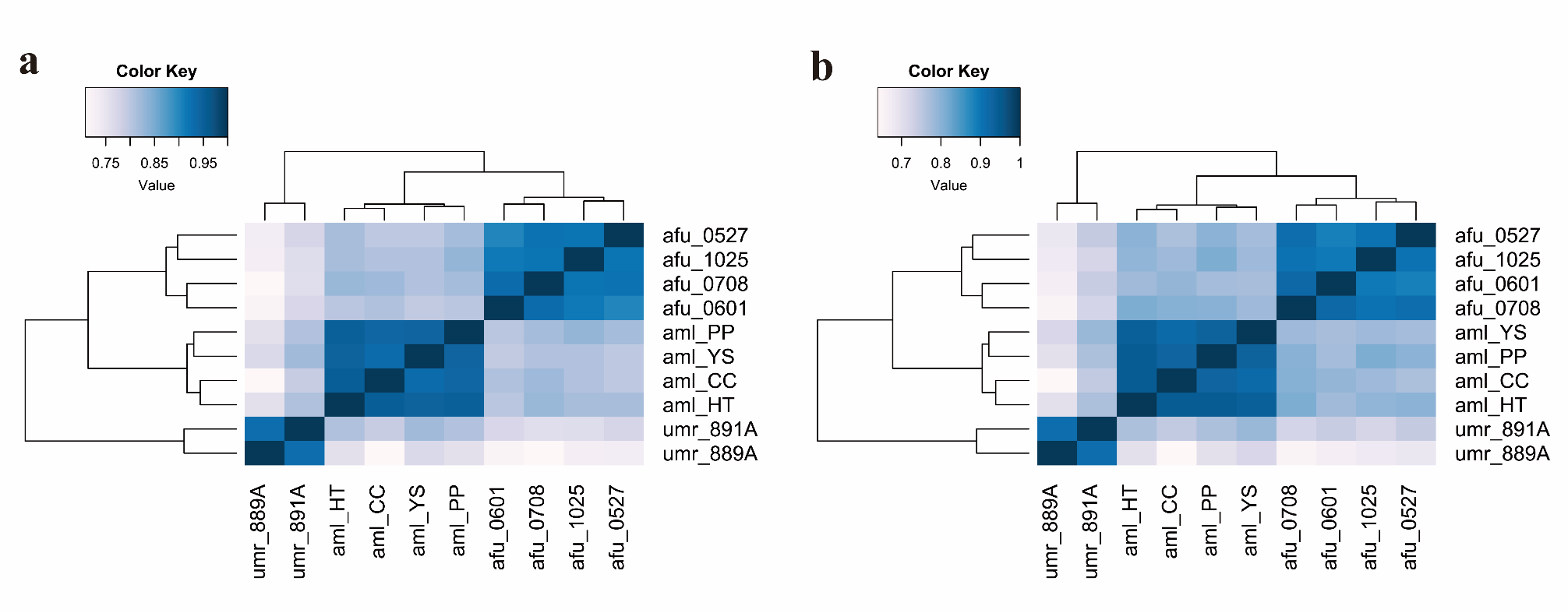


**Fig. S1. Clustering analyses of the modified and unmodified transcriptome datasets. (a)** Clustering analyses of the modified transcriptome datasets. **(b)** Clustering analyses of the unmodified transcriptome datasets. Distance between samples is measured by Spearman's rank correlation coefficient. Abbreviations: Aml: giant panda, Afu: red panda, Umr: polar bear.


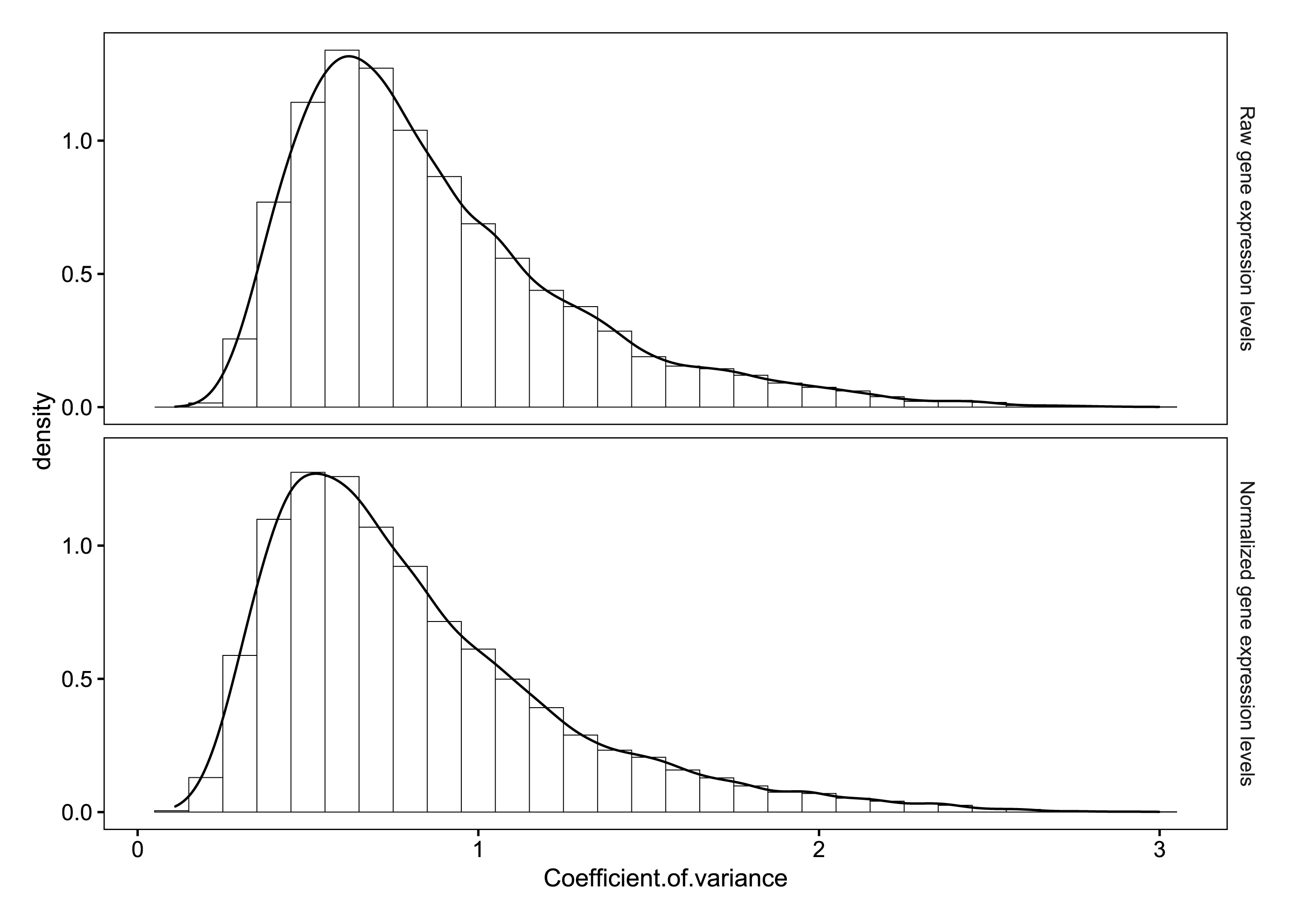


**Fig. S2. Distributions of coefficient of variance of gene expression levels among liver samples before and after normalization, for all 1:1 single-copy orthologues.** A normal density curve is added to the histogram to make the distribution of CV more appealing.


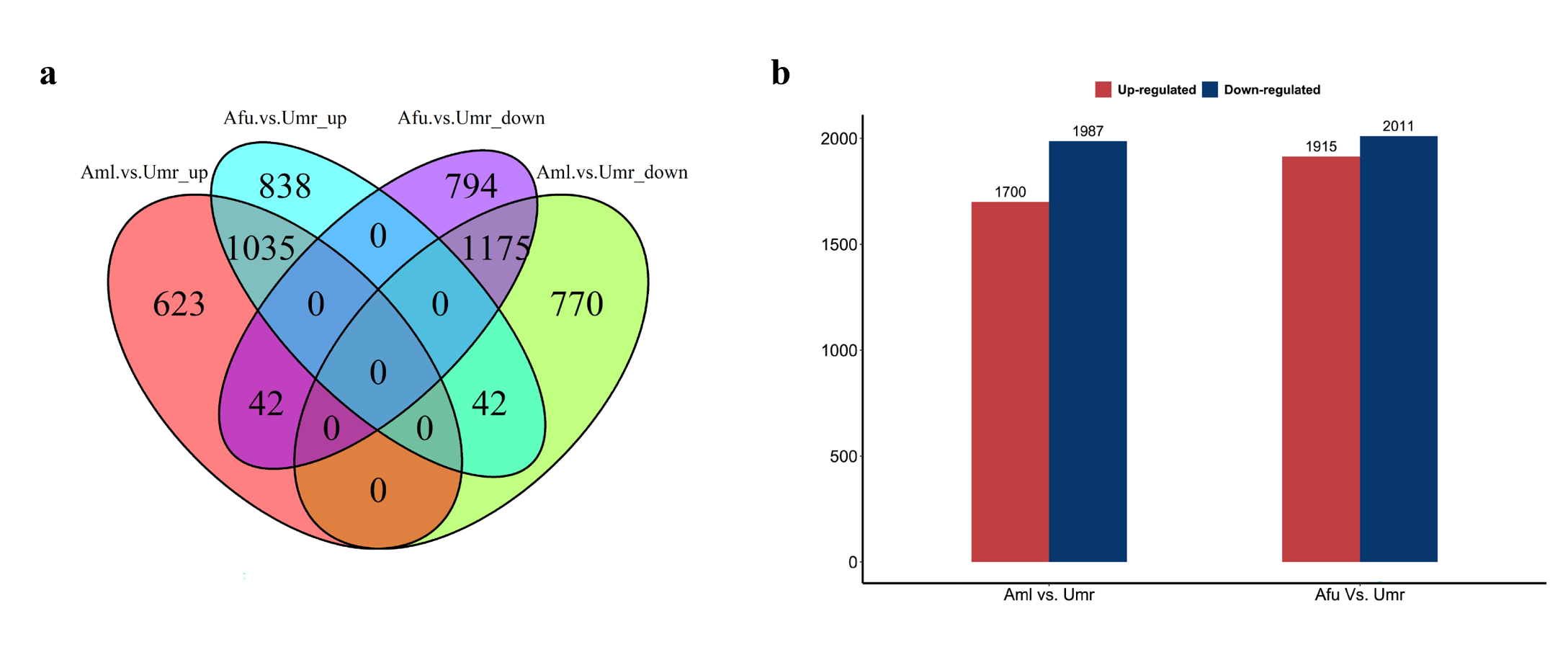


**Fig. S3. The differentially expressed genes (DEGs) in Aml vs. Umr group and Afu vs. Umr group in liver samples.** (a) Venn diagram indicates DEGs that are shared in Aml vs. Umr group and Afu vs. Umr group in liver samples. (b) The number of up- and down-regulated DEGs in Aml vs. Umr group and Afu vs. Umr group in liver samples. Bar plots in red and blue indicate up- and down-regulated DEGs respectively. Abbreviations: Aml: giant panda, Afu: red panda, Umr: polar bear.


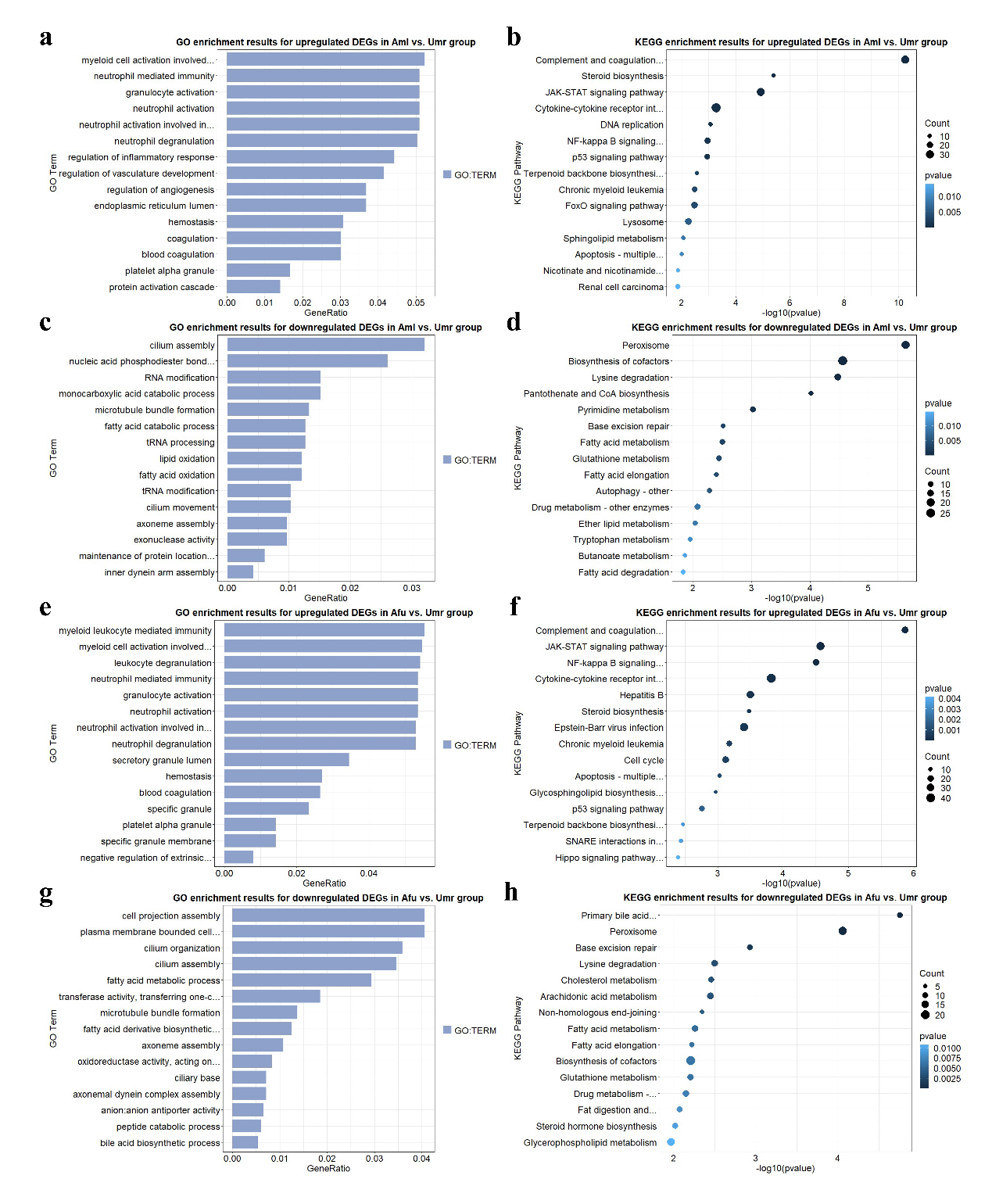


**Fig. S4. Significantly enriched GO categories and KEGG categories of differentially expressed genes (DEGs).** (a) Significantly enriched GO categories for upregulated DEGs in Aml vs. Umr group. (b) Significantly enriched KEGG categories for upregulated DEGs in Aml vs. Umr group. (c) Significantly enriched GO categories for downregulated DEGs in Aml vs. Umr group. (d) Significantly enriched KEGG categories downregulated DEGs in Aml vs. Umr group. (e) Significantly enriched GO categories for upregulated DEGs in Afu vs. Umr group. (f) Significantly enriched KEGG categories for upregulated DEGs in Afu vs. Umr group. (g) Significantly enriched GO categories for downregulated DEGs in Afu vs. Umr group. (h) Significantly enriched KEGG categories for downregulated DEGs in Afu vs. Umr group. The top 15 most significantly enriched items/pathways are shown. X-axis indicates the GeneRatio of the GO enriched items or the -log10(pvalue) of KEGG enriched pathways, Y-axis indicates the name of the item/pathway. The number of genes in the KEGG enriched pathway is indicated by the size of the circle. Abbreviations: Aml: giant panda, Afu: red panda, Umr: polar bear.

**
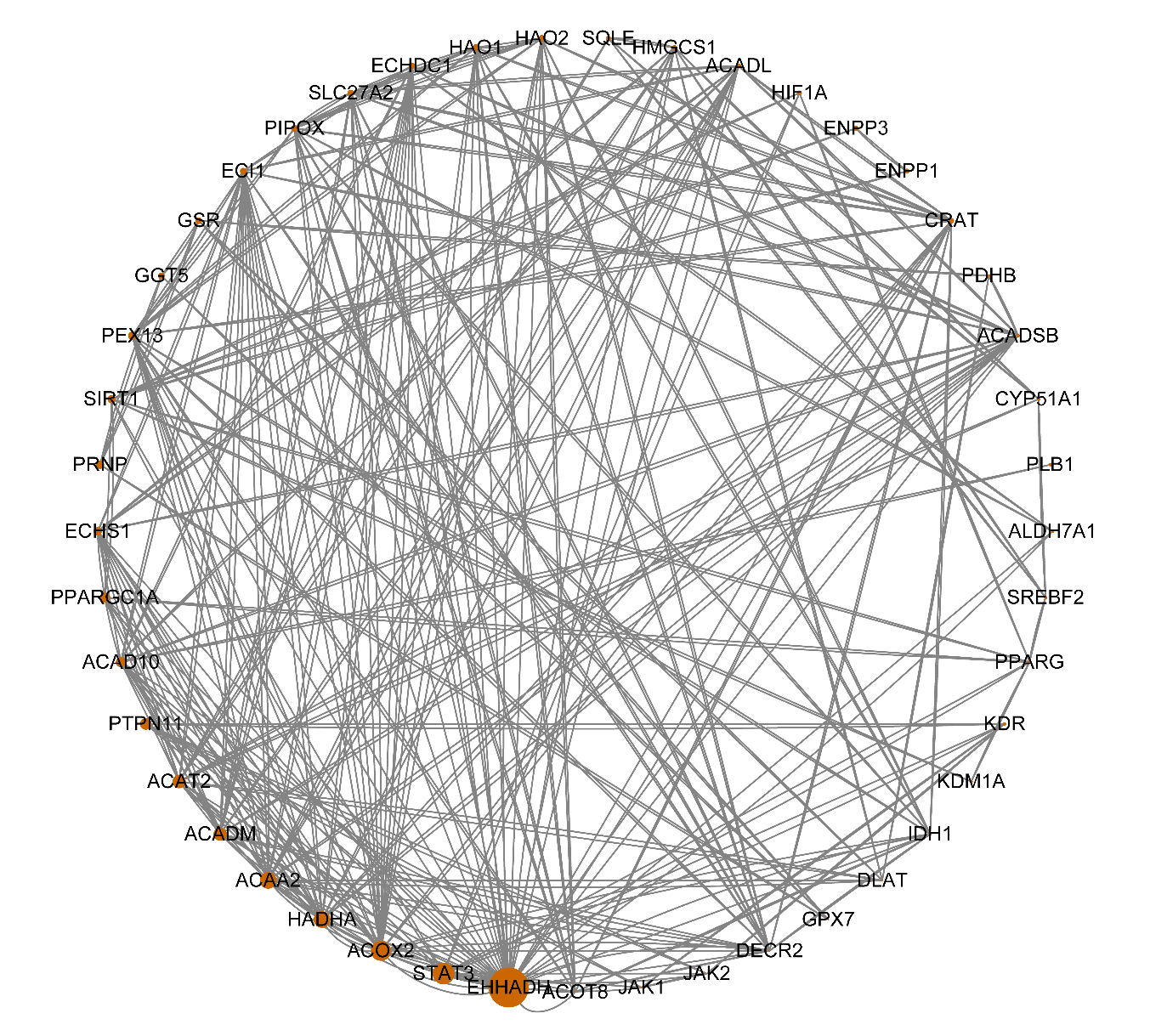
**

**Fig. S5. The network of PPI analysis of metabolism-related differentially expressed genes (DEGs) in Aml vs. Umr group in liver samples.** The sub-network contained hub genes is extracted. The size of the circle represents the degree level of node gene. The bigger the circle, the more degree of node gene. Abbreviations: Aml: giant panda, Afu: red panda, Umr: polar bear.


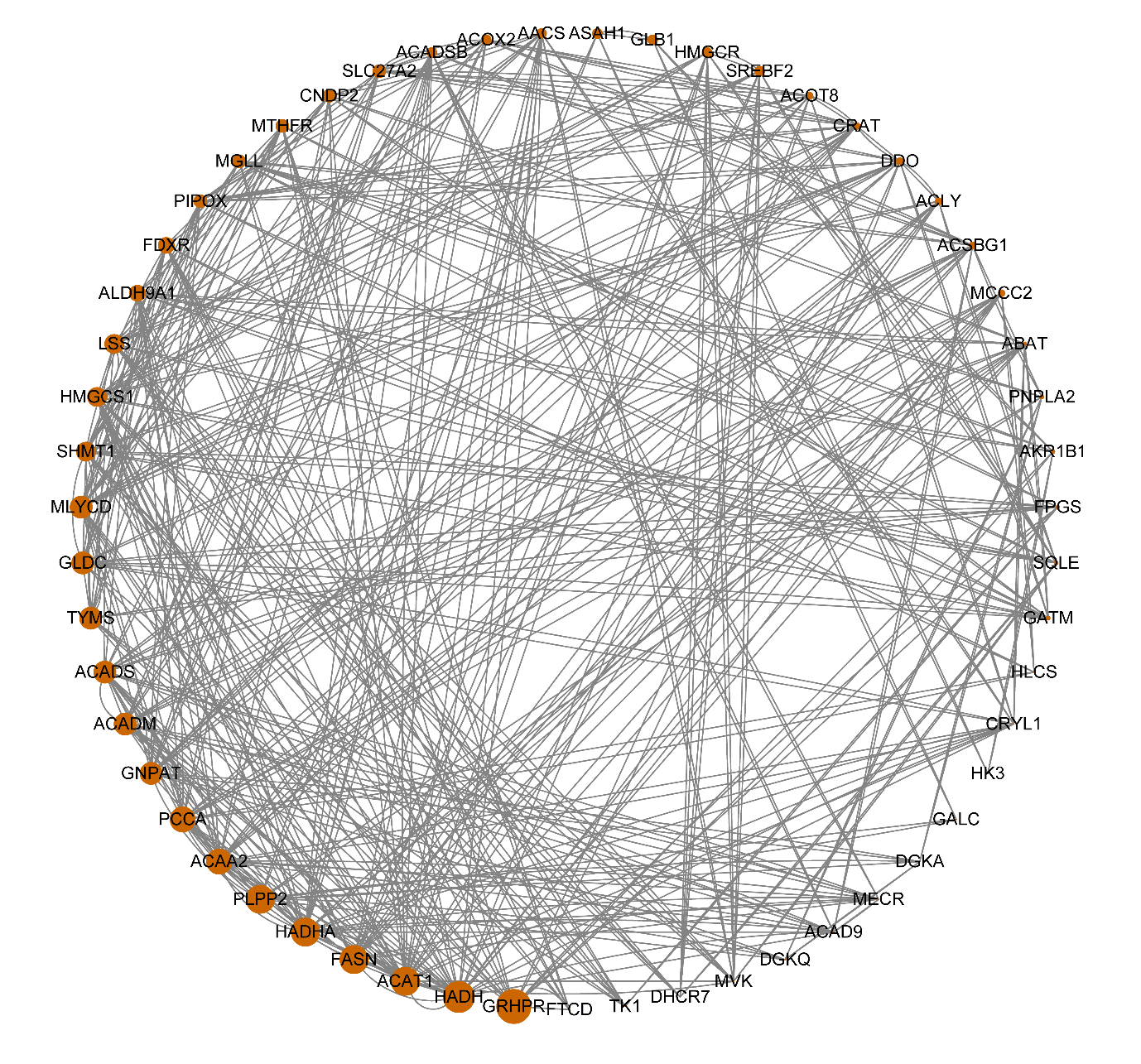


**Fig. S6. The network of PPI analysis of metabolism-related differentially expressed genes (DEGs) in Afu vs. Umr group in liver samples.** The sub-network contained hub genes is extracted. The size of the circle represents the degree level of node gene. The bigger the circle, the more degree of node gene. Abbreviations: Aml: giant panda, Afu: red panda, Umr: polar bear.


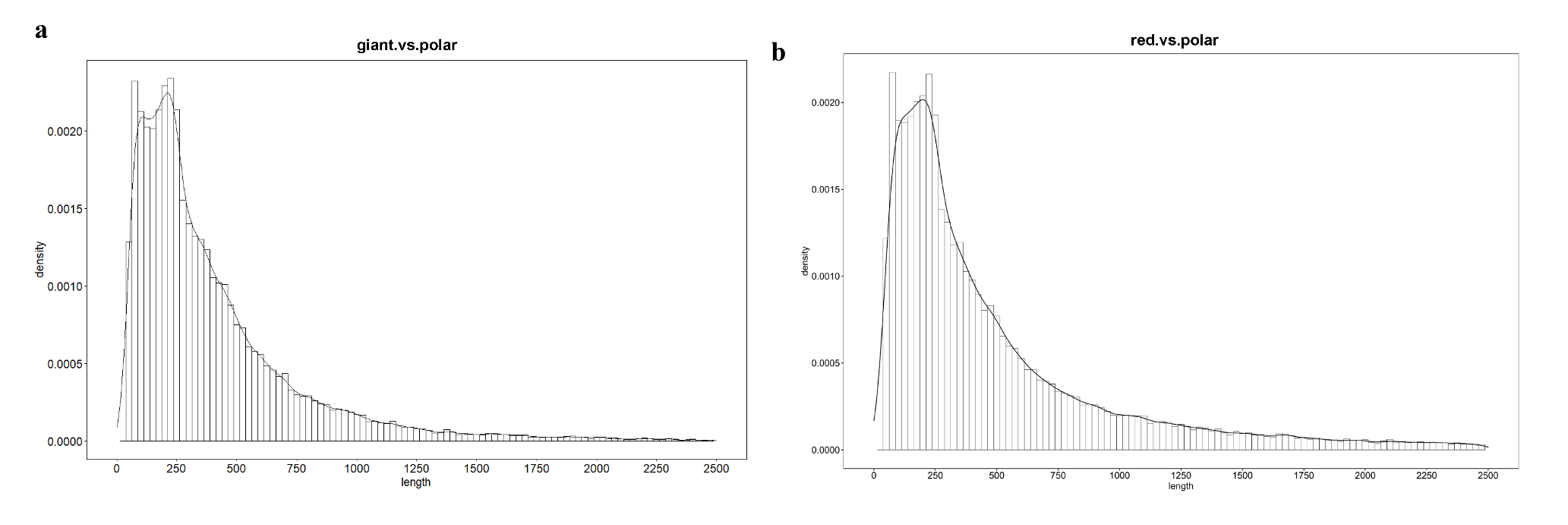


**Fig. S7. Distribution of DMRs in giant pandas vs. polar bears group in liver samples.** (a) Distribution of DMRs in giant pandas vs. polar bears group in liver samples. (b) Distribution of DMRs in red pandas vs. polar bears group in liver samples. X axis indicates the length of DMRs.


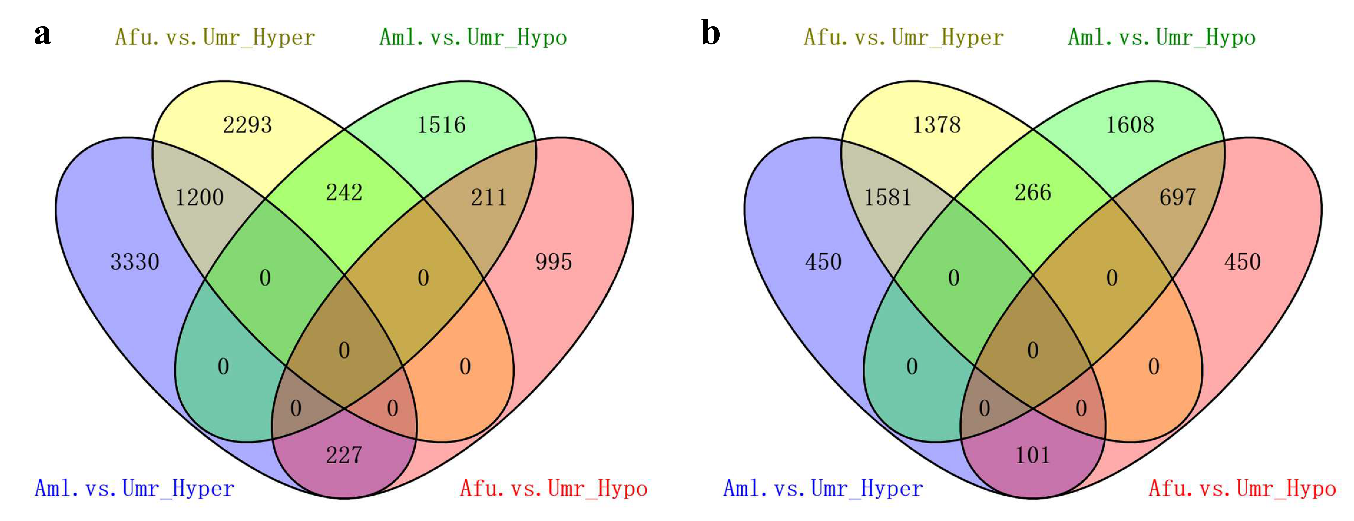


**Fig. S8. The** **differentially methylated genes (DMGs) in Aml vs. Umr group and Afu vs. Umr group in liver samples.** (a) Venn diagram indicates gene-body DMGs that are shared in Aml vs. Umr group and Afu vs. Umr group in liver samples. (b) Venn diagram indicates promoter DMGs that are shared in Aml vs. Umr group and Afu vs. Umr group in liver samples. Abbreviations: Aml: giant panda, Afu: red panda, Umr: polar bear, Hyper: Hypermethylated, Hypo: Hypomethylated.


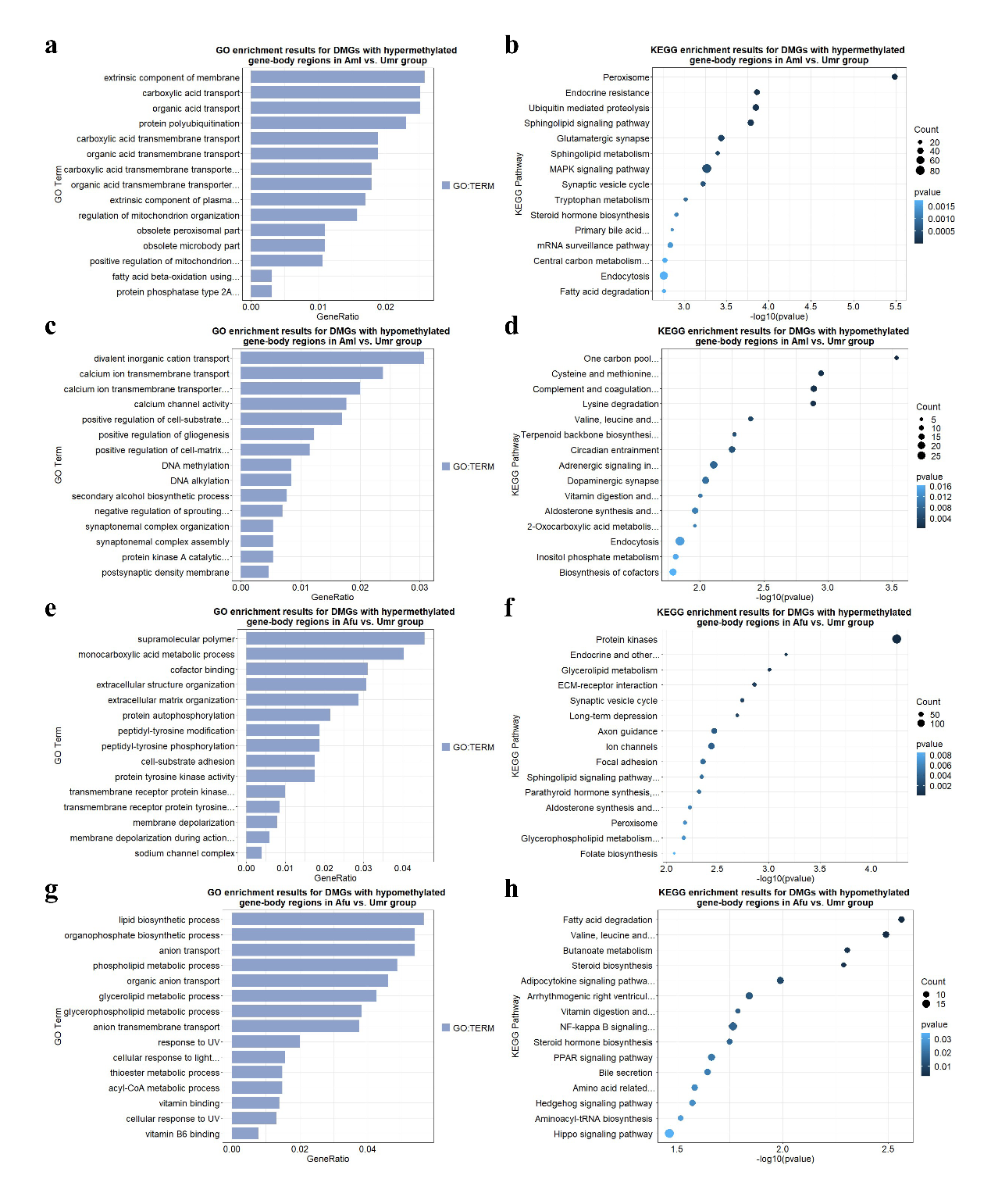


**Fig. S9. Significantly enriched GO categories and KEGG categories of gene-body DMGs.** (a) Significantly enriched GO categories for hypermethylated genes in the gene-body region in Aml vs. Umr group. (b) Significantly enriched KEGG categories for hypermethylated genes in the gene-body region in Aml vs. Umr group. (c) Significantly enriched GO categories for hypomethylated genes in the gene-body region in Aml vs. Umr group. (d) Significantly enriched KEGG categories hypomethylated genes in the gene-body region in Aml vs. Umr group. (e) Significantly enriched GO categories for hypermethylated genes in the gene-body region in Afu vs. Umr group. (f) Significantly enriched KEGG categories for hypermethylated genes in the gene-body region in Afu vs. Umr group. (g) Significantly enriched GO categories for hypomethylated genes in the gene-body region in Afu vs. Umr group. (h) Significantly enriched KEGG categories for hypomethylated genes in the gene-body region in Afu vs. Umr group. The top 15 most significantly enriched items/pathways are shown. X-axis indicates the GeneRatio of the GO enriched items or the -log10(pvalue) of KEGG enriched pathways, Y-axis indicates the name of the item/pathway. The number of genes in the KEGG enriched pathway is indicated by the size of the circle. Abbreviations: Aml: giant panda, Afu: red panda, Umr: polar bear.


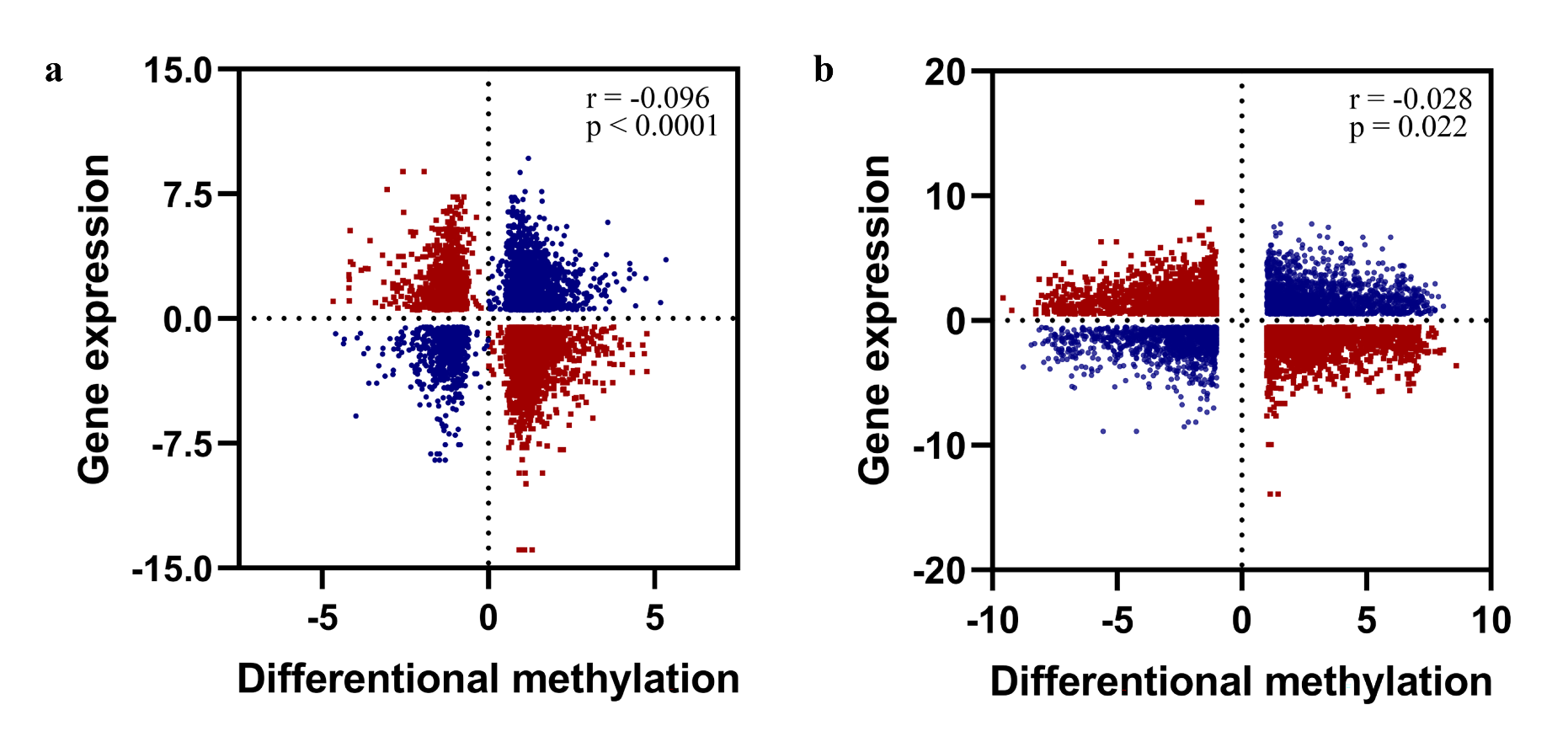


**Fig. S10. Spearman correlation analysis of methylation levels with gene expression in the gene-body region and promoter region.** (a) Spearman correlation analysis of methylation levels with gene expression in the gene-body region. (b) Spearman correlation analysis of methylation levels with gene expression in the promoter region. Red dots indicate genes with negative correlation between differential methylation level and differential expression, blue dots indicate genes with positive correlation between differential methylation level and differential expression, r indicates correlation coefficient, P indicates significance level.
